# Comparative effectiveness of an individualized model of hemodialysis vs conventional hemodialysis: a study protocol for a multicenter randomized controlled trial (the TwoPlus trial)

**DOI:** 10.1186/s13063-024-08281-9

**Published:** 2024-06-28

**Authors:** Mariana Murea, Jochen G. Raimann, Jasmin Divers, Harvey Maute, Cassandra Kovach, Emaad M. Abdel-Rahman, Alaa S. Awad, Jennifer E. Flythe, Samir C. Gautam, Vandana D. Niyyar, Glenda V. Roberts, Nichole M. Jefferson, Islam Shahidul, Ucheoma Nwaozuru, Kristie L. Foley, Erica J. Trembath, Merlo L. Rosales, Alison J. Fletcher, Sheikh I. Hiba, Anne Huml, Daphne H. Knicely, Irtiza Hasan, Bhaktidevi Makadia, Raman Gaurav, Janice Lea, Paul T. Conway, John T. Daugirdas, Peter Kotanko, on behalf of the Two Plus Research Consortium, Denisse A. Funes, Jessica Guillaume, Victoria Shoyelu, Katherine Vergara, Lyn B. Lyman, Fatima Salmi, Erika Adams, Jessica Farrell, Nancy Ginsberg, Christa Howard, Suzanne Shabdue, Shawanna Jackson, Seth Johnson, Randall D. Blackie, Sheetal Chaudhuri, Priya Desai, Kristy Hamilton, Igor Shumilin, Diana Clynes, Valerie Gonzalez, Erin Kahle, Marie Mitchell, Jennifer Rate, Brindusa Burciu, Lilliana Serrano, Alexandra Peluso, Valeria G. Bittencourt, Zohreh Forghani, Elnaz R. Ghalechi, Allison Green, Marina Markovic, Debra Martin, Caroline Poulton, Simran Singh, Katlyn Stiles, Ashleigh Trapuzzano, Joni Baker, Susan Trynosky

**Affiliations:** 1 Department of Internal Medicine, Section on Nephrology, Wake Forest University School of Medicine, Medical Center Boulevard, Winston-Salem, NC USA; 2 Renal Research Institute, New York, NY USA; 3 Department of Foundations of Medicine, Center for Population and Health Services Research, NYU Grossman Long Island School of Medicine, New York, NY USA; 4 Department of Nephrology and Hypertension, Glickman Urological and Kidney Institute, Cleveland Clinic, Cleveland Clinic Lerner College of Medicine of Case Western Reserve University, Cleveland, OH USA; 5 Division of Nephrology, University of Virginia Health System, Charlottesville, VA USA; 6 Division of Nephrology, University of Florida, Jacksonville, FL USA; 7 University of North Carolina (UNC) Kidney Center, Division of Nephrology and Hypertension, Department of Medicine, UNC School of Medicine, Chapel Hill, NC USA; 8 Department of Medicine, Division of Nephrology, Johns Hopkins School of Medicine, Baltimore, MD USA; 9 Division of Nephrology, Department of Medicine, Emory University, Atlanta, GA USA; 10 External Relations and Patient Engagement, Division of Nephrology, Department of Medicine, Kidney Research Institute and Center for Dialysis Innovation, University of Washington, Seattle, WA USA; 11 Home Dialyzors United, Columbia, MO USA; 12 Department of Implementation Science, Wake Forest University School of Medicine, Winston-Salem, NC USA; 13 American Association of Kidney Patients, Tampa, FL USA; 14 Division of Nephrology, Department of Medicine, University of Illinois College of Medicine, Chicago, IL USA; 15 Department of Internal Medicine, Section on Nephrology, LLC Icahn School of Medicine at Mount Sinai, New York, NY USA

**Keywords:** End-stage kidney disease, Hemodialysis, Incremental, Randomized controlled trial

## Abstract

**Background:**

Most patients starting chronic in-center hemodialysis (HD) receive conventional hemodialysis (CHD) with three sessions per week targeting specific biochemical clearance. Observational studies suggest that patients with residual kidney function can safely be treated with incremental prescriptions of HD, starting with less frequent sessions and later adjusting to thrice-weekly HD. This trial aims to show objectively that clinically matched incremental HD (CMIHD) is non-inferior to CHD in eligible patients.

**Methods:**

An unblinded, parallel-group, randomized controlled trial will be conducted across diverse healthcare systems and dialysis organizations in the USA. Adult patients initiating chronic hemodialysis (HD) at participating centers will be screened. Eligibility criteria include receipt of fewer than 18 treatments of HD and residual kidney function defined as kidney urea clearance ≥3.5 mL/min/1.73 m^2^ and urine output ≥500 mL/24 h. The 1:1 randomization, stratified by site and dialysis vascular access type, assigns patients to either CMIHD (intervention group) or CHD (control group). The CMIHD group will be treated with twice-weekly HD and adjuvant pharmacologic therapy (i.e., oral loop diuretics, sodium bicarbonate, and potassium binders). The CHD group will receive thrice-weekly HD according to usual care. Throughout the study, patients undergo timed urine collection and fill out questionnaires. CMIHD will progress to thrice-weekly HD based on clinical manifestations or changes in residual kidney function. Caregivers of enrolled patients are invited to complete semi-annual questionnaires. The primary outcome is a composite of patients’ all-cause death, hospitalizations, or emergency department visits at 2 years. Secondary outcomes include patient- and caregiver-reported outcomes. We aim to enroll 350 patients, which provides ≥85% power to detect an incidence rate ratio (IRR) of 0.9 between CMIHD and CHD with an IRR non-inferiority of 1.20 (*α* = 0.025, one-tailed test, 20% dropout rate, average of 2.06 years of HD per patient participant), and 150 caregiver participants (of enrolled patients).

**Discussion:**

Our proposal challenges the status quo of HD care delivery. Our overarching hypothesis posits that CMIHD is non-inferior to CHD. If successful, the results will positively impact one of the highest-burdened patient populations and their caregivers.

**Trial registration:**

Clinicaltrials.gov NCT05828823. Registered on 25 April 2023.

**Supplementary Information:**

The online version contains supplementary material available at 10.1186/s13063-024-08281-9.

## Administrative information

**Table:** 

**Title {1}**	Comparative effectiveness of an individualized model of hemodialysis vs conventional hemodialysis: a study protocol for a multicenter randomized controlled trial (the Two Plus trial)
**Trial registration {2a and 2b}**	ClinicalTrials.gov identifier: NCT05828823 Registered on 25 April 2023
**Protocol version {3}**	Protocol version 0.5 before recruitment start-up Approved by central IRB on 31-01-2024
**Funding {4}**	Patient-Centered Outcomes Research Institute (PCORI) Award CER-2022C1-26300
**Author details {5a}**	[SPIRIT guidance: Affiliations of protocol contributors.] 1. Wake Forest University School of Medicine 2. Renal Research Institute
**Name and contact information for the trial sponsor {5b}**	Patient-Centered Outcomes Research Institute (PCORI) 1828 L Street NW, Suite 900 Washington, CD 20036 Phone: (202) 827-7700 Email: info@pcori.org
**Role of sponsor {5c}**	The study funder (PCORI) has no role in the design, execution, analyses, interpretation of data, manuscript writing, or decision to submit results for this study.

## Introduction

### Background and rationale {6a}

#### Background

##### Central problem

Patients diagnosed with kidney dysfunction requiring dialysis (KDRD), a condition widely labeled end-stage kidney disease (ESKD), are treated with a conventional “one-size-fits-all” strategy of chronic hemodialysis (HD). The regimen of thrice-weekly HD, known as “conventional HD” (CHD), has been the norm since the 1970s, yet it was implemented without the support of prospective studies to validate the adequacy of this treatment frequency and dose in all patients with new-onset KDRD [1, 2]. Since then, CHD has been compared solely against more frequent HD [3, 4]. Target HD dose per treatment was validated in clinical trials that studied only thrice-weekly CHD in patients with KDRD and *no* residual kidney function [5, 6]; those results were viewed as “optimal” HD for all patients. Initiation of CHD is marked by a sudden rise in hospitalizations and mortality [7] that peak in the first 6 months of HD when adverse events are 3-fold higher than during later dialysis periods [8]. HD induces tissue damage in all organs—including the heart, brain, and gut [9, 10]. The abrupt switch from pharmacologic-based treatment to “full-dose” thrice-weekly CHD is believed to cause systemic circulatory “stress” and multi-organ injury [11], which, in the context of preexisting comorbidities, contribute to proximate adverse outcomes [9, 12]. An alternative to CHD consists of delivering HD at a dose no higher than would be needed to complement existing levels of endogenous residual kidney function while maintaining patient well-being; we call this approach “clinically matched incremental HD” (CMIHD). Patients who may benefit from CMIHD are those who have residual kidney function levels equivalent to one or two HD treatments in urea clearance metrics [13].

##### Patient-identified research priorities

A top priority of patients and other stakeholders is finding ways to reduce HD-related burden [14]. Starting dialysis is stressful to patients and their caregivers, with the initial months being highly critical for both adaptation and mortality [8]. From patients’ and caregivers’ perspectives, the reported stressors of HD are “uncertainty about the future,” “limits on physical activities,” “interference with social activities,” and “interference with job” [15]. Patients report that dialysis-associated fatigue impacts work, home responsibilities, and social participation [16]. In the Empowering Patients on Choices for Renal Replacement Therapy (EPOCH-RRT) study [17] and the Standardized Outcomes in Nephrology–Hemodialysis (SONG-HD) Initiative [18, 19], patients receiving CHD reported that the largest negative impact on quality of life is how their HD schedule interferes with work and school, or how it can change life plans [20]. They felt that dialysis therapy should be more flexible to improve their functioning [20]. Furthermore, patients and caregivers valued the ability to travel, dialysis-free time, and not feeling “washed-out” after dialysis [21].

##### Growing evidence in support of CMIHD

A growing body of studies has shown that incremental transition from pre-dialysis to twice-weekly and then thrice-weekly hemodialysis is safe [22]. Observational studies in patients with incident KDRD and residual kidney function suggested a twice-weekly HD start, compared with thrice-weekly HD, yielding longer or similar [23–28] patient survival when adequate residual kidney function was present. One less HD treatment a week can offer more dialysis-free time, more flexible travel options, increased physical activity, and substantially better health-related quality of life [29, 30]. In addition, residual kidney function, which may be better preserved with incremental HD, is associated with better health-related quality of life [31]. Despite the accumulation of clinical experience and research findings, these have yet to persuade most nephrology professional stakeholders, resulting in the underutilization of incrementally prescribed HD. Consequently, this treatment option remains inaccessible to most patients commencing chronic HD in the USA and numerous other nations, which represents a barrier to patient care choice.

#### Rationale

*Residual kidney function at HD Initiation* national guidelines, updated in 2015, endorses twice-weekly HD for patients with kidney urea clearance (or urea elimination) levels ≥2.0 mL/min [32]. Kidney function at HD initiation, measured as average kidney urea and creatinine clearance from blood, ranges from 7.8 to 9.7 mL/min/1.73 m^2^; >90% of patients [33, 34] have ≥5 mL/min/1.73 m^2^, indicating that many who begin HD still have some residual kidney function [35]. Among patients with incident KDRD, studies showed that about 25–40% would be eligible for initial treatment with twice-weekly HD [34, 36–40]. Yet, national registry data show CMIHD is provided to less than 1% of patients who start HD [24, 41]. Lack of prospective studies to evaluate the clinical effectiveness and operationalization of CMIHD has hindered systematic incorporation of this treatment option into clinical practice.

##### Scope of the problem

Major gaps in CHD paradigm include: (a) it has not been tested in those with new-onset KDRD with residual kidney function [42]; (b) it is not tailored to each patient’s kidney function [43–45]; and (c) it unnecessarily subjects some patients to burdensome and costly dialysis overtreatment [46, 47]. There is a need for rigorous, implementation-effectiveness controlled trials to compare CMIHD and CHD. In the TwoPlus trial, we will address 3 essential questions: (1) Can CMIHD treat clinical symptoms of advanced kidney failure in patients with residual kidney function? (2) Does CMIHD confer better quality of life? and (3) What factors mediate CMIHD implementation, according to different stakeholders?

### Objectives {7}

#### Primary objective

The primary objective is to compare the effectiveness of CMIHD with CHD in patients with new-onset KDRD who have residual kidney function. The primary outcome is the cumulative incidence rate of all-cause death, hospitalizations, or emergency department visits over an average period of follow-up of 2 years.

#### Secondary objective

The secondary objective is to compare patient- and caregiver-reported outcomes and other clinical effectiveness outcomes.

#### Tertiary objective

In parallel, we will characterize intervention implementation using process evaluation to identify contextual factors related to CMIHD implementation.

#### Hypotheses

The primary hypothesis is that, compared to CHD, CMIHD will have similar effectiveness assessed as a composite of all-cause emergency department visits, hospitalizations, or death. After showing noninferiority, we will test whether there are fewer safety events in patients treated with CMIHD.

The secondary hypotheses are that, compared to patients treated with CHD, patients treated with CMIHD will:

(i) Report better quality of life, and caregivers will report less caregiving burden
(ii) Experience better preservation of residual kidney function
(iii) Have similar control of anemia, bone mineral metabolism, and nutrition parameters
(iv) Have fewer vascular access complications
(v) Have improved mental and health outcomes.

The tertiary hypothesis is that the intervention effect size on the primary and secondary outcomes will be moderated by effective implementation as measured by the process evaluation.

### Trial design {8}

**Fig. 1:**
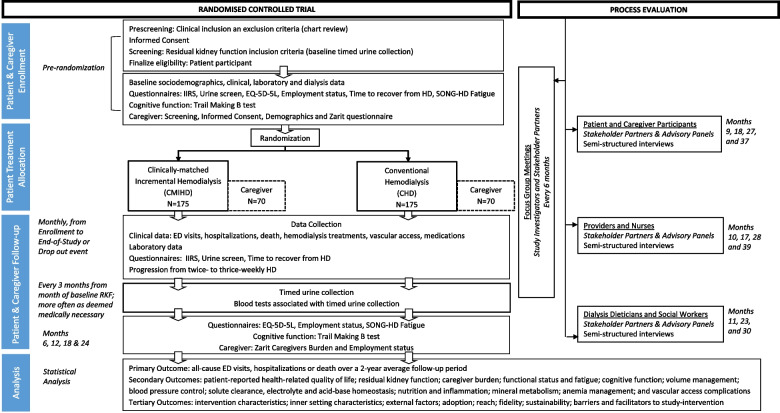
The TwoPlus trial: study design and flowchart of recruitment, treatment allocation, and endpoints. Timepoint of assessment (months) is set from the month of baseline residual kidney function for patients, month of informed consent for caregivers, and Clinical Center recruitment start-up month for process evaluation

The TwoPlus trial is an individually randomized, parallel-group, type 1 hybrid effectiveness-implementation non-inferiority clinical trial with process evaluation (Fig. 1). The study has two concurrent activities:

A. Randomized controlled trial (RCT), to quantitatively assess and compare patient clinical outcomes, patient-reported outcomes, and caregiver-reported outcomes, using randomized treatment allocation, in a 1:1 ratio, to either CMIHD or CHD; and
B. Process Evaluation, to qualitatively assess stakeholders’ (patient participants, caregiver participants, dialysis treating providers, dialysis nurses, dialysis dieticians, and dialysis social workers) views on processes related to individualized HD during study conduct, using semi-structured interviews and focus group meetings.

Patients who provide informed consent for study participation and who met all eligibility criteria will be randomized, in a 1:1 ratio, to one of two HD treatment models (Fig. 1):

- CMIHD (intervention group): 2 HD sessions/week, ≥4 h per each HD session; and adjuvant pharmacotherapy (loop diuretics, potassium binder, and/or sodium bicarbonate). During follow-up, the HD schedule will migrate to 3 HD sessions per week when one or more *Criteria for Progression* are met.
- CHD (control group): 3 HD sessions/week, as prescribed by the treating provider, typically ≥3 h per HD session.

## Methods: participants, interventions, and outcomes

### Study setting {9}

The study will enroll 350 adult patients and 140 adult caregivers (of patient participants) in the USA of America (NCT05828823). Study participants will also include members of a Stakeholder Advisory Panel comprised of diverse stakeholders from all Clinical Centers. The target setting is community outpatient dialysis units and inpatient dialysis units at medical institutions caring for patients with new-onset KDRD [ESKD]. Enrollment will take place at 8 Clinical Centers represented by national healthcare institutions (Additional file 1, Figure S1) and their affiliated outpatient dialysis units. Study oversight will take place from the Clinical Coordinating Center (CCC) and Data Coordinating Center (DCC). Selection of Clinical Centers was determined by considering the patient population, ensuring an adequate volume and sociodemographic diversity for effective study recruitment. Selection criteria also considered the expertise of investigators in clinical trials, availability of research infrastructure, endorsement from regional and national leadership within the affiliated dialysis organization, and the demonstrated engagement of the study team in preparing for the implementation of the study.

### Eligibility criteria {10}

#### Patients

Inclusion criteria are categorized into clinical inclusion criteria and residual kidney function inclusion criteria.

Clinical inclusion criteria

1. Age ≥ 18 years
2. New-onset kidney dysfunction requiring dialysis (also known as end-stage kidney disease [ESKD], end-stage renal disease [ESRD], or chronic kidney disease stage 5 on dialysis [CKD5D]) started on chronic, in-center HD, or anticipated to be started on chronic, in-center HD within the next 6 weeks
3. Has received ≤18 sessions of intermittent HD (i.e., on HD for ≤6 weeks) at the time patient is approached for potential study participation

Residual kidney function inclusion criteria

(4) Kidney urea clearance ≥3.5 mL/min/1.73 m^2^
(5) Urine volume of ≥500 mL/24 h

Exclusion criteria

1. Serum potassium ≥5.8 mEq/L
2. Serum sodium ≤125 mEq/L
3. Serum bicarbonate level ≤17 mEq/L
4. Requirement or anticipated requirement of high-volume ultrafiltration
5. History of medical non-adherence that, in the opinion of the Site Investigators and/or treating provider, precludes safe study participation
6. A medical condition that, in the opinion of the Site Investigators and/or treating providers, would jeopardize the safety of the participant
7. Expected dialysis modality change (e.g., home HD, peritoneal dialysis) or kidney transplantation within the next 6 months
8. Estimated survival of <6 months, in the opinion of the Site Investigators and/or treating providers
9. Estimated transfer to a dialysis facility outside the care of the participating study team within the next 6 months
10. Known pregnancy, or positive serum pregnancy test, or planning to attempt to become pregnant in a woman of childbearing capacity
11. Unable or unwilling to follow the study protocol for any reason

Rationale for thresholds of residual kidney function

Based on a urea kinetics model, total stdKt/V urea clearance (total stdKt/V = dialysis stdKt/V + kidney stdKt/V) of ≥2.10 can be achieved with 2 HD sessions per week, each at a dose similar to thrice-weekly HD (i.e., dialysis spKt/V ≥1.20) when residual kidney urea clearance function is 3.5 mL/min/1.73 m^2^ or above [47]. A more recent model of urea kinetics that gives more weight to residual kidney function indicates that kidney urea clearance levels ≥2.0 mL/min/1.73 m^2^ are sufficient to complement twice-weekly HD [48–50]. Besides kidney urea clearance, 24-h urine volume is a requisite element of adequate residual kidney function [30, 51]. For this study, we selected conservative thresholds for residual kidney function consisting of kidney urea clearance of ≥3.5 mL/min/1.73 m^2^ and urine volume of ≥500 mL/24 h.

#### Caregivers

A patient-identified caregiver(s) will be eligible for study participation if they meet the following eligibility criteria:

- Is of age ≥18 years
- Has self-identified as caregiver (or care partner) for the respective patient participant
- Has one of the following relationships with the patient participant, providing aid to the patient for activities related to daily care or health care:
  i. Is a family relative of the patient: spouse, daughter, son, sister, brother, father, mother, grandchild, brother-in-law, sister-in-law, uncle, aunt); or
  ii. Is a close friend.

(d) Does not have known psychiatric and neurologic disorders (through direct inquiry from the person)*
(e) Is not a member of the medical or healthcare team
(f) Does not provide care for another patient with chronic illness
(g) Has not experienced severe life events within the 3 months prior to enrollment

To be randomized in this study, the candidate patients must meet all the inclusion criteria and none of the exclusion criteria. More information accompanying superscript characters to eligibility criteria is given in the Additional file 2.

#### Advisory panels

The members of the Advisory Panels will be selected to have the following characteristics:

- *Patient and Caregiver Advisory Panel* will be comprised of enrolled patients and caregivers:
  1. Patient enrolled and actively followed in the clinical trial;
  2. Patient randomized to one of the treatment groups;
  3. Caregiver enrolled and actively followed in the study.

- *Dialysis Treating Provider Advisory Panel* will be comprised of treating nephrologists, dialysis medical directors, and/or advanced practice practitioners.
  1. Treating nephrologists
    1. Physician board-certified in Nephrology;
    2. At least 1 year of clinical experience since Nephrology board certification; and
    3. Treating adults with KDRD on HD at one or more dialysis facilities affiliated with the Clinical Center.
  2. Dialysis medical directors
    1. Physician board-certified in Nephrology;
    2. Served as Dialysis Medical Director for at least 1 year at one or more outpatient dialysis facilities affiliated with the Clinical Center.
  3. Advanced practice practitioners (physician assistant, nurse practitioner)
    1. Advanced Practice Practitioner credentialed in Nephrology;
    2. At least 1 year of clinical experience in Nephrology-based practice since credentialed; and
    3. Treating adults with KDRD on HD at one or more dialysis facilities affiliated with the Clinical Center.
- *Dialysis Nurse Advisory Panel* will be comprised of dialysis nurses and/or nurse managers.
  1. Licensed as a Registered Nurse or more advanced credentials; and
  2. Employed as Dialysis Nurse, Dialysis Charge Nurse, and/or Dialysis Nurse Manager for ≥1 year at one or more outpatient dialysis facilities affiliated with the Clinical Center.
- *Dialysis Dietitian Advisory Panel*
  1. Licensed as a Registered Dietitian; and
  2. Employed as a Dialysis Dietitian for ≥1 year at one or more outpatient dialysis facilities affiliated with the Clinical Center.
- *Dialysis Social Worker Advisory Panel*
  1. Licensed as Medical Social Worker; and
  2. Employed as Dialysis Dietitian for ≥1 year at one or more outpatient dialysis facilities affiliated with the Clinical Center.

### Who will take informed consent? {26a}

Informed consent will be obtained from patients, caregivers, and members of the advisory panels to participate in the TwoPlus trial. There will be three types of participant consent:

- Patient informed consent for study participation: in-person and signed informed consent
- Patient participant informed consent for limited data collection: in-person and signed informed consent
- Caregiver consent: telephone consent; waiver of in-person, signed informed consent
- Provider consent: electronic consent for survey, telephone consent for semi-structured interview; waiver of in-person, signed informed consent

More information regarding participants’ informed consent is available in Additional file 3.

### Additional consent provisions for collection and use of participant data and biological specimens {26b}

In the consent form, participants will be requested to allow the utilization of their data in accordance with central and local Institutional Review Board (IRB) privacy policy. In the event of drop-out, data collected to the date of drop-out will be used in statistical analyses of study results. Additionally, participants will be asked for their consent to share pertinent data with the funder. For patients who cannot provide informed consent, the study team will not seek consent from an independent, legally designated representative; these patients will be deemed ineligible to participate in the study. At the time of this writing, the TwoPlus trial does not involve the collection and storage of biological samples.

## Interventions

### Explanation for the choice of comparators {6b}

We will compare incrementally prescribed HD that starts with twice-weekly HD with adjuvant pharmacologic therapy and steps up to thrice-weekly hemodialysis as needed based on changes in residual kidney function levels (CMIHD or intervention group) with conventional HD that starts with thrice-weekly HD (CHD or control group). Adjuvant pharmacotherapy (e.g., loop diuretics, potassium binders, bicarbonate) can enhance residual kidney function and symptom management [34, 52–54]. These drugs, ordinarily prescribed before HD initiation, are well tolerated. Based on international registry data, continuation of loop diuretics after HD initiation was associated with lower weight gain between HD treatments, twice the odds of retaining residual kidney function [55], and more stable blood pressure during HD [53]. Potassium binders can effectively enhance potassium excretion in patients with advanced kidney disease [56], and maintain potassium homeostasis in patients treated with less frequent HD [57, 58] or those on CHD prone to hyperkalemia [59]. Thus, we will include adjuvant pharmacotherapy in the CMIHD group while the patient is on twice-weekly hemodialysis.

### Intervention description {11a}

#### HD prescription

During the study, the HD dose per session will target spKt/V ≥1.20 in both treatment groups [32, 47, 48]. Excluding HD frequency and session duration allocation at randomization, all other HD treatment elements will be prescribed according to the treating providers (Table 1).

**Table 1:** HD prescription according to randomized treatment group^a^

Variable	CMIHD (intervention group)	CHD (control group)
HD sessions per week	2 days until one or more *Criteria for Progression* are met; then 3 days or more often, according to the treating team	3 days or more often, according to treating team
Dialysis urea clearance	spKt/V ≥1.20 per HD session	spKt/V ≥1.20 per HD session
Session duration	≥240 min per HD treatment while on 2 HD/week	Per treating team

^a^Additional elements of HD prescription (e.g., dialysate Na, K, Ca baths; anticoagulation; target weight; anemia and mineral metabolism management) will occur according to local care

#### Blood tests and timed urine collection

These are recommended to be followed during the study in all participants. Per standard care, blood tests are done once or twice a month at outpatient dialysis units in all patients receiving HD. A blood test frequency of twice a month in patients randomized to CMIHD is advised; the lab tests and frequency can be adapted according to clinical judgment by the treating providers and Site Investigators (Additional file 1, Table S1). Timed urine collection will be obtained at a minimum frequency of every 3 months starting from the month of baseline residual kidney function assessment that was obtained at the time of screening and more often as deemed medically necessary by the treating providers and/or Site Investigators.

#### Adjuvant pharmacotherapy in the CMIHD group

The following medications have been termed “adjuvant medications”: diuretics, sodium bicarbonate, and potassium binder. These medications are Food and Drug Administration (FDA)-approved for the medication indication utilized during the study. Most patients are on one or more of these medications before HD treatment. The use of these medications is advised for patients assigned to CMIHD, the decision on the use and dose of these medications is guided by the Site Investigators according to their judgement (Table 2). These medications can also be used (and are often used) in patients treated with CHD according to the judgement of treating providers and/or Site Investigators. Loop diuretics [34, 52–54, 60], potassium binding agent, sodium bicarbonate [61, 62], and/or a potassium binding agent [56, 63] are recommended to those randomized to CMIHD to maintain volume, electrolyte, and acid-base homeostasis [64].

**Table 2:** Adjuvant pharmacotherapy: suggested approach for the TwoPlus trial

**Medication**	**CMIHD (intervention group)**		**CHD (control group)**
Loop +/− thiazide diuretic	*All participants*. Goal to optimize volume status based on interdialytic weight gain and blood pressure		Per treating nephrologist
Sodium bicarbonate	*All participants*. Goal pre-HD serum TCO_2_ level 20–22 mEq/L		Per treating nephrologist
Potassium binder	*When needed*. *At the discretion of the Site Investigators and treating nephrologists.* Goal pre-HD serum K level <5.5 mEq/L		Per treating nephrologist
**Suggested doses of adjuvant pharmacotherapy medications in CMIHD**			
***Medication***	***Clinical context example***	***Suggested treatment***	***Dose titration***
Loop diuretic	On diuretic prior to HD initiation	Double the dose, administer on non-HD days	Based on blood pressure, volume status, or other clinical parameters
	Not on diuretic prior to HD initiation	Start Furosemide (or equivalent^a^) 80 mg/day, non-HD days	
Sodium bicarbonate	Pre-HD serum TCO02 19–18 mEq/L	1300 mg/day, non-HD days	Based on pre-HD serum TCO2 level to keep 20–22 mEq/L
	Pre-HD serum TCO02 ≤17 mEq/L	1300 mg twice per day, non-HD days	
Potassium binder	Pre-HD serum K 5.0–5.4 mEq/L	Patiromer: 8.4 g, once per day, non-HD days Or Lokelma: 5 g, once per day, on non-HD days Or Any FDA-approved potassium binder at dose and frequency recommended by the site investigator and\or treating providers	Based on pre-HD serum K level to keep <5.5 mEq/L
	Pre-HD serum K 5.5–5.8 mEq/L	Patiromer: 16.8 g, once per day, non-HD days Or Lokelma: 10 g, once per day, on non-HD days Or Any FDA-approved potassium binder at dose and frequency recommended by the site investigator and\or treating providers	

^a^Dose equivalence between oral loop diuretics:

Furosemide 40 mg = Torsemide 20 mg = Bumetanide 1 mg

K, potassium; TCO2, bicarbonate

##### Loop diuretic

An oral loop diuretic will be prescribed to all patient participants in CMIHD during the period of twice-weekly HD, to be taken on non-HD days. For those on a loop diuretic before HD initiation, the same loop diuretic will be continued, and the dose will be doubled and administered on non-HD days. Those not on a loop diuretic before HD initiation will be started on a loop diuretic at a dose and frequency deemed medically appropriate; for example, Furosemide 80 mg per day, the dose of Furosemide is expected to range between 80 and 320 mg per day, in one or two divided doses, on non-HD days. Treating providers and Site Investigators may elect not to prescribe a loop diuretic, discontinue administration, or change dose during the study according to medical needs.

##### Bicarbonate buffer

Oral sodium bicarbonate will be prescribed to all patient participants in CMIHD during the period of twice-weekly HD, to be taken on non-HD days. Sodium bicarbonate will be prescribed on non-HD days for patient participants with pre-HD serum bicarbonate level ≤19 mEq/L. In clinical practice, sodium bicarbonate dose and frequency are adjusted based on pre-HD serum bicarbonate level, with a goal of 20–22 mEq/L. Treating providers and Site Investigators retain the discretion to opt against prescribing a bicarbonate buffer, halt its administration, or adjust the dosage as necessary based on medical requirements during the study.

##### Potassium-binding agents

Any potassium binder can be used as deemed medically necessary during the study and at the discretion of the Site Investigators and\or treating providers. It is recommended that, when administration of a potassium binder is deemed medically necessary, the dose and frequency will be adjusted for target pre-HD serum potassium level <5.5 mEq/L.

### Criteria for discontinuing or modifying allocated interventions {11b}

#### Criteria for progression from twice-weekly to thrice-weekly HD

All patient participants will be monitored according to standard care. The treating provider, in collaboration with the site investigators, will decide on progression from twice-weekly to thrice-weekly HD in the CMIHD treatment group. *Criteria for Progression* are summarized in Table 3 as a guidance to providers in decision-making process regarding progress from twice-weekly HD to thrice-weekly HD. Changes in HD prescription in both treatment groups will be at the discretion of the treating providers. The decision to transition from twice- to thrice-weekly HD will be made by the treating nephrologist and/or the site investigator, not by a Research Coordinator. No criterion, taken in isolation, is an absolute indication for transitioning from twice- to thrice-weekly HD. Each criterion will be judged in the overall clinical context for each individual to decide on medical necessity and timing of transition from twice- to thrice-weekly HD. More information accompanying superscript characters to *Criteria for Progression* is given in the Additional file 2.

**Table 3:** Criteria for progression from twice-weekly to thrice-weekly HD*

**Residual kidney function criteria** ^€^
1. Kidney urea clearance <2.5 mL/min/1.73 m^2 #^
2. Urine volume <500 mL/24 h ^#^, despite optimized diuretic regimen
**Clearance criteria by blood tests**
3. Pre-HD serum K ≥5.8 mEq/L ^#^, with or without EKG changes of hyperkalemia, despite treatment with oral K binder
4. Pre-HD serum Na ≤125 mEq/L ^#^
5. Pre-HD serum bicarbonate level ≤17 mEq/L ^#^, despite optimized treatment with oral sodium bicarbonate
6. Unable to attain spKt/V ≥1.20 ^#^
7. Unable to attain total stdKt/V (kidney stdKt/V + dialysis stdKt/V) ≥2.10 ^#^
**Volume management criteria**
8. Inter-dialytic weight gain ≥ 4% of target weight ^#^ , despite optimized diuretic regimen
9. Ultrafiltration rate ≥13 mL/kg/h ^#^, despite optimized diuretic regimen
10. Post-HD residual weight ≥5% of target weight ^#^, despite optimized diuretic regimen
11. Uncontrolled heart failure, which, in the opinion of the treating nephrologist, warrants more frequent HD
12. Uncontrolled hypertension, which, in the opinion of the treating nephrologist, warrants more frequent HD
**Other clinical criteria**
13. EKG abnormalities which, in the opinion of the treating nephrologist, are due to electrolyte and/or acid-base disequilibrium and require more frequent HD
14. Clinical event that requires ≥1 unplanned HD treatment for its resolution, believed to be related to twice-weekly HD
15. Clinical condition, which, in the opinion of the treating nephrologist, warrants more frequent HD
16. Non-adherence to timed urine collection for ≥2 consecutive occasions when timed urine collection was recommended by the treating providers and/or Site Investigators
17. Intravenous antibiotic administration required thrice-weekly with HD ^¥^
18. New condition rendering the patient with inability to perform timed urine collection (e.g., stroke, urinary incontinence)

*EKG* Electrocardiogram, *HD* Hemodialysis, *spKt/V* Single pool Kt/V, *stdKt/V* Standard Kt/V

Information accompanying superscript characters is found in the Additional file 2

#### Censoring events

The occurrence of any of the events below will lead to censoring of patient participation:

- Participant withdraws consent and does not allow continued collection of limited data
  - Reason for consent withdrawal will be documented.
- Withdrawal of HD by the treating team due to kidney function recovery
  - Reason for withdrawing the participant, i.e., kidney function recovery and dialysis is discontinued, will be documented.
- Patient undergoes kidney transplantation, and hemodialysis is discontinued.
- Transition to peritoneal dialysis and patient participant does not allow continued collection of limited data.
- Transition to home HD and patient participant does not allow continued collection of limited data.
- Diagnosis of pregnancy
- Drop-out event

It is noted that “Withdrawal of HD by the treating team as an end-of-life event” is not a censoring event. If and when “Withdrawal of HD” represents a scenario of HD withdrawal as part of hospice care, data is collected until end-of-study event, such as death.

#### Drop-out events

The occurrence of any of the events below will be logged as drop-out event, and data collection will be censored at the time of event occurrence:

- Transfer of nephrology care outside participating health system network
- Lost to follow-up

#### End-of-study events

These events indicate a study finishing point with no further data collection. They include:

- Participant death
- Censoring events
- Study end date

#### Temporary patient participant status change

For scenarios when the patient participant travels (e.g., vacation, visiting) and will be absent from receiving HD at the participating healthcare system, data collection will be temporarily suspended if the temporary status change is no longer than 6 weeks. Upon return to the dialysis unit/healthcare system participating in the study, the patient will continue to be a study participant, and data collection will be resumed. During the period of patient participant temporary status change, data collection from affiliated caregiver participant will be temporarily held and resumed as soon as patient participant data collection resumed.

#### Caregivers and duration of follow-up

Caregivers will be followed in the study until one of the following end-of-study events that pertain to caregiver participation:

- Caregiver withdraws consent
- Death of the caregiver
- Caregiver is lost to follow-up
- The affiliated patient participant had an end-of-study event, and data are no longer collected for the patient participant

#### Temporary caregiver participant status change

For scenarios when the caregiver participant travels (e.g., vacation, visiting), data collection will be temporarily suspended if the temporary status change is no longer than 6 weeks. Upon return, the caregiver will continue to be a study participant, and data collection will be resumed. During the caregiver participant temporary status change, data collection from affiliated patient participant will continue to be collected.

### Strategies to improve adherence to interventions {11c}

#### Non-adherence events

Non-adherence to serial urine collection, transitioning from twice- to thrice-weekly HD, and/or crossover between groups will be monitored. The following will be logged as non-adherence events:

1. Non-transition to thrice-weekly HD from twice-weekly HD in patients in the CMIHD treatment group when the treating providers make this recommendation to the patient.
2. Patient’s decision to get HD twice-weekly when they have been randomized to the CHD treatment group and the treating provider recommends thrice-weekly HD.
3. Patient has not submitted timed urine collection on one or more scheduled month(s) of assessment.
4. Missed HD treatments (not due to hospitalization)

Collected data will be summarized as:

- *N* (%) patients in CMIHD who refused to progress to thrice-weekly HD when recommended
- *N* (%) patients in CHD who crossed over to twice-weekly HD
- Average percentage of missed HD treatments (not due to hospitalization) per month in the CMIHD group
- Average percentage of missed HD treatments (not due to hospitalization) per month in the CHD group

#### Approach to optimizing adherence to study-related processes of care

To mitigate the potential for non-adherence events, the research team will apply the following measures: (i) training and education of the research team and local providers on the study score and study-related processes of care before study recruitment begins; (ii) thorough sharing with the prospective patient participant of information and expectations on adherence to the study protocol when obtaining informed consent for study participation; (iii) establishing close lines of communication between the local study teams and the treating providers teams and dialysis personnel; and (iv) providing quarterly Patient Participant Report.

#### Training

Integration of CMIHD into usual care is a complex process, and thus, education of local providers is a prerequisite to initiating study enrollment. Training will occur in three levels, in a train-the-trainer format. First, level, the research staff of the CCC and DCC will conduct training modules to educate the Site Investigators and the Research Coordinators on study procedures, safety monitoring and adverse event adjudication, data collection, data entry, assembly of Advisory Panels during the study, local lines of communication, and local workflows and procedures to ensure study interventions are adequately implemented. Training of the Research Coordinators will also include acquisition of knowledge on HD, vascular access, residual kidney function, and workflows at outpatient dialysis units. Simulated case scenarios will be used to optimize the integration of residual kidney function-related tests into local workflow and usual blood tests. All training modules will be video recorded and centrally stored with access to research team members from all Clinical Centers. Second, the Site Investigators will educate the local treating providers (physicians and advanced practice practitioners), dialysis administration, and dialysis personnel on study-related procedures and patient monitoring. The Site Investigators will educate the Research Coordinators in navigating healthcare systems and dialysis electronic medical records (EMRs) to ensure all data needed for this study is optimally collected. Third, the local treating providers will establish their workflows and communication with dialysis personnel as it relates to study intervention implementation.

#### Lines of communication between the study team and treating providers and dialysis personnel

Following randomization, the assigned treatment group (CMIHD or CHD) will be conveyed by the study team to the patient participant, dialysis personnel, and the treating providers (physician and advanced practice practitioners). The communications will consist of elements related to the allocated treatment group, including HD frequency, HD treatment duration, and adjuvant pharmacotherapy, depending on the treatment group. During the study, the study teams will maintain continued communication with the treating providers and dialysis personnel to monitor residual kidney function tests, blood tests, and recommended changes in HD prescriptions and medications.

#### Thorough patient information on study-related processes of care

Prospective patient participants will be given thorough information, education, and expectations associated with study participation. Strong and kind emphasis will be placed on the expectation of serial timed urine collection, the temporary nature of twice-weekly HD, and importance of adherence to dialysis.

### Relevant concomitant care permitted or prohibited during the trial {11d}

Participation in this study will not interfere with any medically indicated procedure or intervention during the study. The decisions and recommendations to start chronic HD will be made by the treating physicians, independent of this study. Participation in this study will not affect treating KDRD (or ESKD) associated comorbid conditions, including the management of anemia, mineral and bone disorders, vascular access, nutritional support, and others, at the discretion of the treating physicians. A patient or caregiver participant may be withdrawn from the study at any time without negative consequences to their clinical care. While an investigator may remove a study participant from study participation at any time, this will not mean that the clinical team will stop HD or clinical treatment of the patient.

### Provisions for post-trial care {30}

There is no provision for post-trial care. The outcome will be determined at the end of the study. However, since participants are routine clinical patients at participating medical institutions, they will continue to receive dialysis treatments and all clinical care as medically required.

### Outcomes {12}

#### Primary outcome

The primary outcome will be a composite of all-cause ED visits, hospitalizations, or death over a 2-year average follow-up period (Table 4).

**Table 4:** Primary and secondary outcomes and measures

**Outcome**	**Measures**	**Level of analysis**	**Data sources**	**Timepoints**
**Primary Outcome**				
Safety	All-cause ED visits, hospitalizations or death	Patient	Patient’s EMR (dialysis and hospital EMR)	Enrollment to end-of-study event
**Main Secondary Outcomes**				
Health-related quality of life	^a^*Illness Intrusiveness Rating Scale*	Patient	Patient	Baseline^b^ and monthly
	EuroQOL-5D-5L score	Patient	Patient	Baseline^b^ and semi-annually^c^
	Employment status	Patient & Care partner	Patient & Care partner	
	Hospital-free days per 100 patient-days	Patient	Patient’s EMR (dialysis and hospital EMR)	Enrollment to end-of-study event
Residual kidney function	^a^*Change* in urine output (mL/24 h)	Patient	Patient	Baseline^b^ and every 3 months or more often when needed
	Change in kidney urea clearance (mL/min/1.73 m^2^)	Patient	Patient	
	Change in kidney creatinine clearance (mL/min/1.73 m^2^)	Patient	Patient	
**Other Secondary Outcomes**				
Care partner burden	Zarit Caregivers Burden Scale	Caregiver	Caregiver	Baseline and semi-annually
Functional status and fatigue	Time to recover from HD	Patient	Patient	Baseline^b^ and monthly
	SONG-HD Fatigue score	Patient	Patient	Baseline^b^ and semi-annually^c^
Cognitive function	Trail Making Test Part B score	Patient	Patient	Baseline^b^ and semi-annually^c^
Volume management	Interdialytic weight gain; Residual weight; Ultrafiltration rate (mL/kg/hour)	Patient	Patient’s EMR (dialysis and hospital EMR)	Baseline^b^ and monthly
Blood pressure control	Average pre- and post- dialysis blood pressure; Number of prescribed antihypertensive agents	Patient	Patient’s EMR (dialysis and hospital EMR)	Baseline^b^ and monthly
Solute clearance, electrolyte and acid-base homeostasis	Total stdKt/V; Pre-HD serum sodium, potassium, and bicarbonate	Patient	Patient’s EMR (dialysis and hospital EMR)	Baseline^b^ and monthly
Nutrition	Serum albumin concentration; Normalized protein catabolic rate	Patient	Patient’s EMR (dialysis and hospital EMR)	Baseline^b^ and monthly

^a^Main secondary outcomes

^b^Pre-randomization

^c^Semi-annually: months 6, 12, 18, and 24 of study participation. EMR, electronic medical record

#### Secondary outcomes

The secondary outcomes will compare the effects of CMIHD and CHD on 13 domains: (i) patient-reported health-related quality of life; (ii) changes in residual kidney function; (iii) hospital-free days per 100 patient-days; (iv) caregiver burden; (v) functional status and fatigue; (vi) cognitive function; (vii) volume management; (viii) blood pressure control; (ix) solute clearance, electrolyte and acid-base homeostasis; (x) nutrition and inflammation; (xi) mineral metabolism; (xii) anemia management; and (xiii) vascular access complications. Domains (i) and (ii) are the main secondary outcomes (Table 4) because they are linked with our overarching hypothesis regarding CMIHD effectiveness on patient-centered outcomes and the primary outcome.

##### Patient- and caregiver-reported outcomes

We will capture patient-reported outcomes and caregiver burden using instruments validated in patients with KDRD. The following instruments will be completed at baseline (pre-randomization) and during follow-up (study month): Illness Intrusiveness Rating Scale (IIRS) [65] (monthly), EuroQOL-5 Dimensions-5 Level [66], and employment status [67] (months 6, 12, 18, and 24). All interviewers will be trained in proper questionnaire management and report collection. Baseline patient data will be collected in person (after obtaining informed consent for study participation and before randomization). During follow-up, trained research personnel will collect data in person (for cognitive and physical function assessment), in person, or by telephone (for questionnaires). Caregiver burden will be assessed using the Zarit Caregiver Burden Scale [68]. Baseline caregiver data will be collected at the time of informed consent and before patient randomization. Patient-reported functional status and fatigue will be assessed using the Time to recover from HD [69] and SONG-HD Fatigue [70] instruments.

##### Changes in residual kidney function

Urine volume and kidney urea and creatinine clearance will be measured no less often than every 3 months and within 2–4 weeks of hospitalization in both groups. At enrollment, dialysis personnel, treating providers, and/or trained research personnel will instruct patient participants on performing timed urine collections using uniform instructions. Urine collections will be analyzed at the lab used by the dialysis center. Results will be entered in the centralized database to calculate kidney urea clearance per week (i.e., kidney stdKt/V) [71] and automatically sent to the Site Investigators.

##### Hospital-free days per 100 patient days

We define hospital-free days as all days alive that are spent outside of an acute-care hospital, long-term acute-care hospital (LTACH), or in an emergency department (ED), including days spent wholly or in part under “observation” status. All other days, including days spent in a long- or short-stay nursing facility, inpatient hospice facility, or rehabilitation facility, count as hospital-free, as would all days at home, including those with home-based medical services. This definition aligns with how others have operationalized days alive and outside the hospital [72, 73].

##### Other outcomes

Patient participants’ cognitive function will be assessed using change in Trail Making Test Part B score [74]. Volume management, solute clearance, electrolyte and acid-base homeostasis, and measurement related to those outcomes will be collected and analyzed. Conversion rates will be compared from in-center HD to home dialysis, kidney transplantation, and dialysis withdrawal. Components of the primary outcome will be compared as cause-specific and time-to-event outcomes between the groups.

##### Process evaluation

These are outcomes concerning the evaluation of processes related to this study (Table 5). Process evaluation will include (a) *Intervention characteristics*, to assess organizational readiness to change [75], intervention acceptability and appropriateness [76]; (b) *Inner setting characteristics*, to assess barriers and facilitators to the adoption of HD intervention at the partnering organizations [77, 78]; (c) *External factors* [79, 80] that mediate implementation; (d) *Adoption*; (e) *Reach*; (f) *Fidelity*, to assess adherence to serial timed urine collection and HD treatment schedule; and (g) *Sustainability*, to assess barriers and facilitators to maintaining intervention. To assess these processes, we will employ surveys and semi-structured interviews with patient participants, caregiver participants, and dialysis stakeholders.

**Table 5:** Process evaluation

**Outcome**	**Measures**	**Level of analysis**	**Source**	**Timepoints**
Reach	Number (%) of patients with incident KDRD eligible for study participation Number (%) of eligible patients who consent for study participation	Patient	Study administrative record (REDCap)	March 2024 to Feb 2026
Adoption	Number (proportion) of participants and participating dialysis units with >90% intervention fidelity	Patient	Patient’s EMR	Enrollment to end-of-study event
Intervention characteristics, Inner settings characteristics, Adaptation & Sustainability	Semi-structured interviews	Patient and Caregiver	Patient & Caregiver Advisory Panel	Months 9, 18, 27, and 37
		Provider	Dialysis Treating Provider Advisory Panel Dialysis Nurse Advisory Panel	Months 10, 17, 28, and 39
			Dialysis Dietitian Advisory Panel Dialysis Social Worker Advisory Panel	Months 10, 17, 28, and 30
Intervention Fidelity	Fidelity Monitoring Tool	Patient	Patient’s EMR	Monthly starting March 2024

*EMR* Electronic medical record. Anticipated enrollment start and end dates are March 2024 and Feb 2026, respectively. Timepoints (Months) for process evaluation are calculated from the month of recruitment start for each Clinical Center

The assessment timepoints for secondary outcomes and process evaluation are listed in Tables 4 and 5, respectively.

### Participant timeline {13}

A flowchart and a schematic diagram of the schedule are presented in Fig. 1 and Table 6, respectively (information accompanying superscript characters in Table 6 and participant timeline is present in the Additional file 2). Semi-structured interviews with members of the advisory panels will occur on a pre-established timeline with months of interviews calculated from the enrollment start-up month for each Clinical Center.

**Table 6:** Time schedule of assessment for patients and caregivers enrolled in the TwoPlus trial

**Category**	**Assessment**	**Study month**				
		**M0**	**M6**	**M12**	**M18**	**M24**
			**M30**	**M36**	**M42**	**M48**
***Prescreening and Screening: Patient and Caregiver***						
Patient	Prescreening: Clinical inclusion and exclusion criteria	x				
	Informed consent	x				
	Urine screen questionnaire	x				
	Trail Making Test B	x				
	Screening: Baseline residual kidney function	x				
Caregiver	Informed consent, Socio-demographics, Employment status, Zarit scale					
***Pre-Randomization: Patient***						
Questionnaires	Illness Intrusiveness Rating Scale	x				
	Time to recover from a dialysis session	x				
	EuroQoL-5 Dimensions-5 Level	x				
	SONG-HD Fatigue	x				
Clinical	Socio-demographics	x				
	KDRD Etiology and Comorbid conditions	x				
	In-center and home medications and dose	x				
Laboratory	eGFR pre-KDRD (most recent prior to HD initiation)	x				
	Labs pre-ESKD: chem, hematology (most recent prior to HD initiation)	x				
Dialysis	Initial HD, as prescribed by treating team (when applicable)	x				
	Vascular access for HD †	x				
	Labs pre-HD: chem, hematology (most recent, after HD was started)	x				
	Target weight, Pre-HD weight, Post-HD weight, UF rate (mL/kg/h)	x				
	Pre- and Post-HD BP and HR, nadir BP and HR, peak BP and HR	x				
	spKt/V, nPCR	x				
***Post-Randomization: Patient Follow-up***						
Clinical	ED visits without hospitalization		*Monthly*			
	Hospitalizations		*Monthly*			
	Death		*Monthly*			
	In-center and home medications and dose		*Monthly*			
Laboratory & Dialysis	Timed urine collection, Residual kidney function assessment		*Every 3 months*			
	HD treatments: prescribed and delivered		*All treatments*			
	Vascular access: used for HD, complications		*Dec, Mar, Jun, Sept*			
	Attendance of HD treatments; Shortened HD treatments by ≥15 min		*Dec, Mar, Jun, Sept*			
	Labs pre-HD: chem, hematology^b^		*Monthly*			
	Target weight, Pre-HD weight, Post-HD weight, UF rate (mL/Kg/h)		*Dec, Mar, Jun, Sept*			
	Pre- and Post-HD BP and HR, nadir BP and HR, peak BP and HR		*Dec, Mar, Jun, Sept*			
	spKt/V, dialysis stdKt/V, renal stdKt/V, total stdKt/V, nPCR		*Dec, Mar, Jun, Sept*			
Questionnaires	Urine screen questionnaire		*Monthly*			
	Illness Intrusiveness Rating Scale		*Monthly*			
	Time to recover from HD		*Monthly*			
	EuroQoL-5 Dimensions-5 Level^c^		x	x	x	x
	Employment status^c^		x	x	x	x
	SONG-HD Fatigue^c^		x	x	x	x
Cognitive function	Trail Making Test Part B		x	x	x	x
***Caregiver Follow-up***						
Questionnaires	Employment Status, Zarit scale		x	x	x	x

Timepoint of assessment (months) is set from the month of baseline residual kidney function for patients, and month of informed consent for caregivers. *BP* Blood pressure, *GFR* Glomerular filtration rate, *HD* hemodialysis, *HR* Heart rate, *KDRD* Kidney dysfunction requiring dialysis, *UF* Ultrafiltration, *spKt/V* Single pool (or per HD treatment) urea Kt/V, *stdKt/V* Standard (or per week) urea Kt/V, *UF* Ultrafiltration

Information accompanying superscript characters is found in the Additional file 2

### Sample size {14}

#### Power considerations for the primary outcome

The sample size of 350 total (175 per treatment group) was calculated under the following assumptions and conditions: (a) reported combined 2-year incidence of all-cause mortality, all-cause ED visits not leading to hospitalization, and all-cause hospital admissions from the ED is 2.954 per person-year for CHD [81]; (b) clinical trial participants tend to be healthier than the overall population with KDRD [82]; thus, our null safety rate will assume a conservative downward adjustment in all-cause mortality estimate by 50% and all-cause ED/hospitalization visit estimate by 30% to yield an incidence rate of 2.021 events per person-year with CHD; (c) a lower incidence rate with CMIHD, i.e., IRR equal to 0.9 [64, 83]; (d) safety events follow a negative binomial distribution using a restricted likelihood estimation variance calculation method and overdispersion equal to 0.12 [57]; (e) 20% dropout rate across groups; (f) average of 2.06 years of HD per participant; (g) non-inferiority margin for IRR of 1.20, corresponding to an unacceptable worsening of safety event rate of 2.426 or higher per person-year in the CMIHD group; (h) ≥85% power; and (i) one-sided level of significance equal to 0.025. This sample size is conservatively adjusted for the healthier population incidence rate of 2.021 events per person-year as suggested in (b); if we use the unadjusted 2-year safety event rate of 2.954 per person-year in (a) for our null safety rate, our sample size of 350 would have >93% power to detect the same non-inferiority margin of 1.20 (Additional file 1, Table S2).

#### Power considerations for secondary outcomes

We calculated statistical power based on a 2-year average follow-up, assuming an analysis of covariance model (ANCOVA) [84], which provides an intuitive interpretation that is less dependent on the number of intermediary measurements. We also assume a correlation of 0.8 between baseline and follow-up values based on preliminary data [57, 66, 68, 83, 85]. Our calculations show detectable standardized effect sizes of 0.201 and 0.233 for 80 and 90% power, respectively, based on an average sample size of 280 participants across the five assessments (baseline and semi-annual for 2 years) (Additional file 1, Table S3). There is no consensus on the appropriate type I error level for secondary outcomes or even whether they should be tested when the primary endpoint is not statistically significant [86–88]. However, for completeness, with a strict Bonferroni correction for the two main secondary outcomes, the detectable effect sizes become 0.221 and 0.253 for 80 and 90% power, respectively, leading to slightly higher detectable average differences for the secondary outcomes.

### Recruitment {15}

All patients initiating chronic HD and undergoing treatment at participating Clinical Centers and the associated outpatient dialysis facilities will undergo a three-stage evaluation to determine their eligibility for the study: prescreening, invitation to participate in the study, and screening (Additional file 1, Figure S2). During the prescreening stage, the research team will assess the patients’ EMRs to check eligibility against inclusion criteria 1 through 3, exclusion criteria 1 through 9, and exclusion criterion 10 when applicable. When needed, the team will consult the patients’ healthcare providers to ascertain eligibility based on exclusion criteria 4 through 9. Patients meeting prescreening conditions will be approached by a member of the study team and receive a comprehensive explanation of the study’s scope and procedures, followed by an assessment against exclusion criteria 10 (where relevant) and 11. During the informed consent process, the patients will be asked about their willingness to participate in semi-structured interviews should they meet all eligibility criteria and be enrolled in the study, and whether they have a caregiver that could be approached for study participation in the form of completing questionnaires and potentially engaging in semi-structured interviews. Should the patients demonstrate a clear understanding of the study’s requirements and provide their written informed consent, they will progress to the screening stage and will be asked to complete a baseline timed urine collection; a serum pregnancy test will be obtained when applicable. Once patients successfully clear the screening process, their caregivers will be approached to participate in the study (Additional file 2). These caregivers will be screened with targeted questions, and upon providing verbal informed consent, they will be asked to complete baseline questionnaires. This step will take place prior to the randomization of the associated patient.

For *process evaluation*, study participants include patients and caregivers enrolled in the study, as well as advisory panels. Patient and caregiver participants from each Clinical Center will form the Patient and Caregiver Advisory Panel (*n*=4–8 patients and *n*=4–8 caregiver participants per interview meeting, from each Clinical Center). Dialysis stakeholders comprised of treating providers including nephrologists, dialysis medical directors, and advanced practice practitioners (*n*=4 per interview meeting, from each Clinical Center), dialysis nurses including nurses and nurse managers (*n*=4 per interview meeting, from each Clinical Center), dialysis dieticians (*n*=2 per interview meeting, from each Clinical Center), and dialysis social workers (*n*=2 per interview meeting, from each Clinical Center) will form Dialysis Treating Providers Advisory Panel, Dialysis Nurse Advisory Panel, Dialysis Dietician Advisory Panel, and Dialysis Social Worker Advisory Panel, respectively.

#### Sampling strategy for members of the advisory panels

For semi-structured interviews, we will aim equal engagement of patient participants from each treatment group and caregivers linked to patients assigned to each treatment group. To help ensure generalizability of experiences, we aim to engage stakeholders of different genders, age groups, races, and socioeconomic status.

We anticipate patient and caregiver enrollment period will span 2 years. The average follow-up period is estimated to be 2 years, with an anticipated range of 1 to 4 years. The unit of randomization will be eligible patients who consent to participate in the study. The unit of analysis will be all study participants.

## Assignment of interventions: allocation

### Sequence generation {16a}

Randomization will be centralized using computer-generated randomly permuted blocks to ensure balance in treatment allocation within each stratum. Randomization will be stratified by clinical center and type of vascular access used (central venous catheter or arteriovenous access) at enrollment.

### Concealment mechanism {16b}

Allocation concealment will be ensured. The study teams will not have access to the randomization until the patient has passed all eligibility criteria and completed all baseline measurements.

### Implementation {16c}

Before proceeding with patient randomization, we will gather all baseline questionnaires from both patients and their caregivers. Once randomization is complete, the assigned treatment group will be communicated to the patient, dialysis staff, and the patient’s nephrology care team, including physicians and advanced practice practitioners, by the Site Investigator.

#### Screen failures of baseline residual kidney function

In the event that patients’ baseline residual kidney function does not satisfy inclusion criteria 4 and 5, they will be notified and excluded from taking part in the study. There are occasions where recorded levels of residual kidney function might be artifactually or temporarily low, such as instances where patients acknowledge incomplete urine collection or have experienced an acute illness. Under these circumstances, the Site Investigator has the discretion to authorize a repeat of the timed urine collection for screening purposes to determine whether the patient qualifies for inclusion based on their baseline residual kidney function.

#### Serum pregnancy test

Women ≤55 years old without a history of hysterectomy will undergo serum pregnancy testing as a component of their eligibility assessment for study participation. This test may be either qualitative or quantitative. Should the pregnancy test result positive, the participant will be ineligible for study participation. For women who become part of the study, routine and serial serum pregnancy tests will not be required by the protocol. Instead, future tests will be administered on an individual basis, aligning with usual care when clinical indications arise.

Assignment of interventions: blinding

### Who will be blinded {17a}

This is an open-label study. Blinding of the participants, dialysis personnel, and clinicians is not feasible; however, data collection for the primary endpoint will be based on objective events. The data analysts in charge of statistical analyses will access to the database after data cleaning and database lock and perform analyses according to a predetermined statistical analysis plan.

### Procedure for unblinding if needed {17b}

The study is open label. Therefore, unblinding is not required.

## Data collection and management

### Plans for assessment and collection of outcomes {18a}

Data collection forms will be provided by the CCC to all participating Clinical Centers.

#### Data collection methods

All data variables related to prescreening, informed consent, screening, questionnaires, timed urine collection and related urine and blood tests, hospitalizations (detailed by reason, duration, dialysis treatments received, and any instances of mechanical ventilation), emergency department (ED) visits (including cause), and deaths (along with cause) will be prospectively and systematically collected on a monthly basis through reviews of healthcare system EMRs, dialysis EMRs, and participant reports, and will be entered in a centralized electronic data capture (EDC) TwoPlus study platform. Patient information routinely logged at outpatient dialysis units within the dialysis EMRs will undergo de-identification and be digitally transmitted to the DCC on a scheduled basis.

Semi-structured questionnaires will be guided by the CFIR (Consolidated Framework for Implementation Research) [89] and RE-AIM (Reach, Effectiveness, Adoption, Implementation, Maintenance) [90, 91] and will be conducted by members of the Implementation Science Team (IST) with experience in qualitative research and conducting semi-structured interviews. These interviews will take place either via telephone or a video platform. The semi-structured interviews will consist of open-ended, qualitative questions that allow and encourage participants to elaborate as they like, which we expect to yield rich personal narratives and provide insight into processes of care and experiences related to the study intervention. The interviews will be audio recorded, professionally transcribed verbatim, and checked for accuracy against the corresponding recording. The IST will also hold periodic (about every 6 months) focus group meetings with Site Investigators and Stakeholder Partners to gather operational adaptations and other perspectives related to the implementation of incremental-start HD.

In events where a participating patient expresses the desire to withdraw from the study, they will be courteously inquired if they permit the continuation of data collection through the non-intrusive gathering of clinical events and relevant variables present within the healthcare system and dialysis EMRs up to the study’s conclusion. If a caregiver participant asks not to be contacted again regarding the study, this will be noted, and they will not be contacted again.

### Plans to promote participant retention and complete follow-up {18b}

#### Pragmatic study design

Research suggests that enhancing retention and reducing missing data can be achieved through several strategies: employing brief assessments, implementing reminders for participants, offering incentives, limiting the number of visits exclusive to the study, and making use of data collection reports [92]. We have integrated these effective methods into our existing strategy to optimize patient engagement and data quality. We will administer succinct participant-reported outcomes that patients complete in approximately 10 min monthly, while caregivers do so semi-annually, and an extended 25-min version for patients every 6 months. Both patients and caregivers can conveniently respond to the questionnaires via telephone. We will integrate semi-annual cognitive assessments into patients’ outpatient dialysis schedules, obtaining Trail Making Test B assessments before the afternoon or after the morning HD sessions. There will be no study-specific visits or needle sticks, integrating all necessary procedures with HD treatments. To optimize compliance with timed urine collections, patients will be given telephone reminders and will be remunerated to incentivize completion. Patients will be advised to bring their collected urine samples to their dialysis sessions for convenience. The use of EMR-extracted outcome data will reduce the risk of disproportionate data loss for participants who are minimally engaged. Disengaged patients wishing to withdraw will be queried for consent to continue collecting their EMR data for research purposes. Finally, the information we will obtain during the semi-structured interviews with the patient participants, caregivers, and dialysis personnel will be used to improve processes of care associated with this recruitment and retention in this study.

#### Additional strategies to improve or monitor adherence to the study protocol

These will include reports generated by DCC:

- Monthly recruitment reports of patients screened, enrolled, and consented (accrual figures)
- Prescreen, informed consent, and screen fails to assess for bias in inclusion/exclusion decisions
- Monthly reports detailing data received at the data center, data consistency, missing data, performance measures, and adherence to the study protocol
- Participant adherence to the assigned treatment group and HD prescription
- Supplementary blinded reports requested by the study investigators or subcommittee that do not disclose allocation-group–specific outcomes (primary, secondary, or any safety outcomes)

### Data management {19}

#### Centralized calculations of residual kidney function and dialysis clearance

The centralized TwoPlus database platform is programmed with validated formulas [71] to calculate residual kidney function, kidney weekly urea clearance (i.e., kidney standard Kt/V [kidney stdKt/V]), dialysis single pool urea clearance (spKt/V), and dialysis weekly urea clearance (dialysis stdKt/V), ultrafiltration rate, residual weight, and inter-dialytic weight gain (Additional file 4). Data collected at the site level (for residual kidney function) and electronically transferred from dialysis EMR to the centralized study platform will be linked using participant identification number (PID), and results will be distributed to the study teams on a regular basis.

#### Data quality

The DCC and CCC will undertake data quality checks, supported by close liaison with the Site Investigators and Research Coordinators at each Clinical Center. Staff at each Clinical Center will be trained to ensure that data are captured reliably using Case Report Forms pro forma as mock data for data entry training in the EDC platform. Ranges for data variables will be placed in the EDC system to reduce the risk of inaccurate data entry. The DCC will issue monthly reports detailing data completeness and omissions, segmented by team and Clinical Center, to Site Investigators and Research Coordinators. These reports will inform biweekly discussions at research meetings aimed at monitoring follow-up rates and addressing data gaps. The DCC will alert the Research Coordinators to any data that are missing or inaccurate.

#### Data security

Wherever possible, all personal and research data will be entered and stored only in electronic and password-protected format. When it is necessary to store personal or research data in hard copies, for example, where there is no access to a laptop or where staff complete paper versions of an encounter, data will be stored at the Clinical Center in a designated base and a locked filing cabinet. Electronic copies of study participant’s personal and study data will be stored on secure shared drives at each Clinical Center working area of the study team. All study data will be password-protected using a password known only to the study team. No study participant’s personal or study data will be downloaded or stored on individual employee’s drives or desktops. Data will be entered into the TwoPlus EDC platform and REDCap. It is Good Clinical Practice (GCP) and 21 CFR Part 11 compliant, with a full audit trail and database lock functionality. The linkage between personal information and routine data collected from individual participants will be securely maintained in a separate, password-protected file to ensure privacy and confidentiality. Audio recordings of semi-structured interviews will be maintained by the Implementation Science Team on a secure, shared drive and stored in an anonymized and encrypted form. Data will not be shared with anyone outside of the study teams and organizations hosting the study. Research data will be archived at each clinical center and centrally for all centrally collated electronic data and stored for a 10-year duration in line with the funders’ and local regulatory policies. After 10 years, research data will be shredded, deleted, or destroyed using confidential data destruction measures in place for each organization of the TwoPlus Research Consortium.

### Confidentiality {27}

The TwoPlus trial will be conducted in accordance with the principles of the Declaration of Helsinki, the International Conference on Harmonization Good Clinical Practice (ICH GCP), applicable United States (US) Code of Federal Regulations (CFR), and institutional regulatory policies at each participating organization. To ensure privacy, a participant identification number (PID) in the form of an alpha-numeric ID for each patient will be assigned to preserve personal information and contacts. Participants’ data will be stored in a secure password-protected file at each clinical center. The drive will only be accessible to the study team members. In both hard and electronic versions, personal and study data will be kept separate. Study data will be identified using PID. This ID will be linked to the participant’s name in a master recruitment log file that will be password-protected, with password known only to the study team.

### Plans for collection, laboratory evaluation, and storage of biological specimens for genetic or molecular analysis in this trial/future use {33}

As of the date of this document, there are no plans for storage of biological specimens.

## Statistical methods

### Statistical methods for primary and secondary outcomes {20a}

#### Primary outcome analysis

The *primary outcome* will be analyzed as the incidence rate of all-cause ED visits not leading to hospitalization, all-cause hospitalizations, or all-cause death for each treatment group. This incidence rate in each HD treatment group will be calculated as the total number of primary outcome events divided by the number of person-years in the study. A participant could have recurring ED visits, hospitalizations, and/or die during the study; all events will be included. Negative binomial regression will be used to model the number of events observed in each group using a dummy variable to denote treatment groups [93]. The time between randomization and the last assessment for each patient will be used as an offset. This model will be adjusted for sex, race, baseline comorbidities, and stratification variables (i.e., Healthcare System, vascular access, and age at enrollment). Adjustments will be made for the potential correlation between patients from the same Clinical Center using generalized estimating equations [94–96]. We will estimate the incidence rate ratio (IRR) in the CMIHD group vs the CHD group as a linear contrast on the log scale, which will then be exponentiated. A one-sided 97.5% confidence bound will be computed, and non-inferiority will be met if that bound is less than 1.20 times the incidence rate in the CHD group. If non-inferiority is established, we will test whether CMIHD is superior to CHD in reducing the IRR of the primary outcome using a fixed sequential approach. Superiority will be claimed if the upper limit of the same 97.5% confidence interval (CI) was <1, with a type I error rate of 0.05 for a two-sided test [97].

#### Secondary outcomes analysis

Changes from baseline in patient- and caregiver-reported outcomes, employment status, and lab values will be analyzed using repeated-measures analysis of covariance (RMANCOVA) [98], starting with an unstructured covariance matrix to account for measurements that are not independent. For the main secondary outcomes, measured monthly, we will use mixed-effects models using baseline value as a covariate and accounting for within-subject and within-clinic center correlation [99, 100]. Taking advantage of the randomization, we will constrain the pre-randomization intervention-specific outcome means to be the same [101]. For randomized trials, constrained mixed-effects models can provide more efficient estimates of post-randomization treatment differences when either baseline or post-randomization measures are missing. In addition to the dummy variable to denote the intervention groups, the model will contain terms for recruitment center, vascular access, and baseline value of the outcome of interest. Interaction effects between the intervention and time will be tested using likelihood ratio tests, while treatment effects will be estimated and tested using linear contrasts. For outcomes collected from caregiver participants, we expect randomization of patient participants would balance caregiver characteristics between groups. A unique identification number that links caregiver data with patient participant data will be created.

The analysis for hospital-free days per 100 patient days will be performed under the intention-to-treat principle and will be measured from the date of randomization to the date of an end-of-study event or end of the study, whichever comes first. For each participant, the primary outcome will be the cumulative number of days that the patient was not hospitalized. We will develop an algorithm that gives different “weight” to a hospitalization event based on the presence or absence of mechanical ventilation and, in later events, based on the number of days of mechanical ventilation. We will compare outcomes between the treatment groups using Poisson regression, modeling the number of hospital-free days as the dependent variable with a log link, the treatment assignment as the predictor, and the natural log of the number of at-risk days as an offset. Covariate adjustment will be performed. Analyses will take into account events of recurring hospitalizations after hospitalization-free days. Deviance residuals and the overall deviance measure will be calculated to assess the overall goodness of fit of the model. If we observe overdispersion, we will explore the negative binomial models and conduct model diagnostics.

##### Potential occurrence of social desirability bias and its effect on patient-reported outcome measurements

Some may argue that since patients in either CMIHD or CHD group are not blinded to randomized intervention, patients in the CMIHD may give more favorable responses to survey questionnaires than patients in the CHD group. This phenomenon has been termed “social desirability bias.” Survey questionnaires will be administered pre-randomization at the baseline visit and then either monthly or semi-annually, depending on the instrument. Baseline responses will not be affected by the intervention. Surveys administered post-randomization could potentially be influenced by treatment assignment, especially the first questionnaire filled after randomization. However, it can be argued that the “optimism” effect related to CMIHD may subside over time. Similarly, patients who transitioned from twice- to thrice-weekly HD may become more pessimistic. To explore these effects, we will look for non-linear association between time on study and current intervention (CMIHD on twice-weekly HD, CMIHD transitioned to thrice-weekly HD or CHD) on the survey scores. We will also describe this as a potential limitation in our papers, as this potential for bias cannot eliminated from the study because blinding is not practical.

##### Analyses for components of primary outcome

When interpreting the findings from clinical trials with composite endpoints, it may be difficult to ascribe efficacy to individual components of the composite, even when overall findings are positive [102]. One way to address this situation is by using shared parameter models, in which a latent variable is included in the parameterization of event rates for individual conditions, and the intervention effect on this variable is assessed. A related approach involves multivariate survival models in which incidence times of multiple endpoints are assessed [103]. An alternative is to follow analyses of the composite with formal tests for heterogeneity in intervention effects on the individual components [104]. While power may be limited due to a small number of events, this approach is designed to detect situations when there are marked differences in how the intervention relates to the various components of the composite primary outcome.

#### Analysis populations

##### Primary and secondary outcomes

Analyses of the primary and secondary outcomes will be conducted as intention-to-treat (ITT), i.e., all randomized participants will be included in primary analyses. This will include all the scenarios when patient participants provide signed informed consent for continued data collection of a limited set. Participant data will be analyzed according to their randomized treatment, regardless of adherence to the prescribed HD schedule in each treatment group.

##### Sensitivity analyses

Sensitivity analyses will be conducted in the per-protocol population set. These analyses will include participants who exhibited full adherence to as-recommended HD treatment schedules. The per-protocol analyses will censor the data on participants who did not adhere to conversion from twice- to thrice-weekly HD and patients with non-adherence and crossover from thrice- to twice-weekly HD. This censoring will apply at the time the first non-adherence is observed. Only data prior to non-adherence will be used in the per-protocol analysis. Analyses will be adjusted by the inverse probability of censoring. Zero-inflated mixed-effects negative binomial models will be fit to assess the effect of time‐varying non-adherence weights. We will (i) use information on recorded AEs and recommended HD prescription; (ii) collect interim-diagnosed comorbidities for those who are medically advised to transition from CMIHD to CHD; and (iii) define each participant when for the first time (in days after randomization), for how long, and how often they were nonadherent to the HD prescription. Propensity score weighted analyses (1/P(adherence)) will be performed.

##### Secondary analyses

These analyses will include an evaluation of crossovers from twice- to thrice-weekly dialysis and as-treated analyses.

##### Analysis plan to account for crossover timing from twice-weekly to thrice-weekly HD

An important analytic question is how to account for the transition (crossover) from twice- to thrice-weekly HD. To do so, we will collect the time of the transition and a second set of “baseline” characteristics data at the time of transition. Patients who transition to thrice-weekly HD will be followed until the end of the observation period. We note that transition would follow loss of residual kidney function (disease progression) such that a two-stage adjustment method can be applied in this context. However, unlike current practice in oncology trials where patients are transitioned from the standard of care to the experimental treatment (because of benefit), patients will be transitioned from the intervention to the “control,” CHD group. Under the two-stage model, treatment effects will be estimated separately for patients who remained on the twice-weekly HD, those who transitioned to thrice-weekly HD, and those who were initially randomized to thrice-weekly HD. These estimates will then be combined to generate an overall treatment effect [105, 106].

##### As-treated analyses

The as-treated analysis will take into account randomized treatment allocation and time-varied receipt of the two HD treatment schedules. The as-treated population will comprise the ITT population and statistical modeling of HD schedule in each treatment group, thus adjusting for the effects of lack of adherence to conversion from twice- to thrice-weekly HD or presence of crossover from thrice- to twice-weekly HD. The as-treated analysis with time‐varying non-adherence weights will track and adjust for pre-randomization and post-randomization prognostic factors that can impact both, adherence to assigned treatment and the primary outcome.

#### Analysis of process evaluation

We will analyze results by thematic analysis [107]. Following the principle of constant comparison, transcripts will be analyzed as interviews are conducted. First-cycle codes will be derived directly from the data [107]. Codes will consist of a short phrase generated by the researcher that captures the essence or attributes of a data segment [107]. We will use open coding and will apply codes to data sections that the analyzing researcher deems appropriate. We will use constant comparison to examine the data, both within a given interview and across interviews. We will use NVIVO qualitative data analysis software to analyze the data and record codes. When a substantial number of interviews (~8) has been completed, we will begin searching for themes by analyzing the initial codes to determine how the codes can be grouped into themes. Themes are constructs that succinctly capture an important pattern in the data in relation to the research question [107]. We will review the candidate themes and read the data extracts for each theme to determine if the data fit the candidate themes. If not, we will then decide if certain data extracts fail to fit the theme or if the theme needs to be reworked. If the data do fit the candidate theme, then we will assess the accuracy with which the set of themes reflects the meanings and relationships of the whole data set [107]. We will identify and revise themes simultaneously as the interviews continue until data saturation. As the themes mature, we will determine how the themes fit together to tell the overall narrative [107]. We will ensure there is minimal overlap between themes and identify any sub-themes that may be contained within a given theme.

##### Triangulation and data synthesis

Our process evaluation is not designed to make immediate changes to the study unless ethical issues require them; early semi-structured interviews and intervention fidelity evaluations will be reported to the study teams and may be considered in intervention implementation. The triangulation protocol aims to produce meta-themes that cut across individual methods [108]. To enhance rigor and trustworthiness, we will conduct the study and report our findings in accordance with the Consolidated Criteria for Reporting Qualitative Research (COREQ) [109]. We will ensure credibility by attaining prolonged engagement in each interview and continuing interviews until data saturation is reached. We will repeat interviews if/as needed to clarify or expand upon any issues not completely explored in initial interviews. We will analyze interviews as they are completed and use constant comparison to test for data saturation. To increase internal validity, interviews will be audio recorded, professionally transcribed verbatim, and checked for accuracy against the corresponding recording. Each interview will be analyzed by at least 2 investigators. To maximize credibility, we will use analytic triangulation, whereby researchers analyze independently and discuss any discrepancies in their codes to arrive at an agreed-upon conclusion. To minimize bias, each interviewer will record field notes regarding details of the interview, the participant, the setting, and any thoughts or impressions before and after the interviews emphasizing self-reflexivity (the process by which investigators become aware of their own biases). To optimize confirmability, we will record memos to create an audit trail of all coding decisions.

### Interim analyses {21b}

Not applicable. There are no interim analyses planned.

### Methods for additional analyses (e.g., subgroup analyses) {20b}

We will perform subgroup analyses for the primary outcome and main secondary outcomes to explore whether the treatment effect is consistent across subgroups [110]. Using the ITT population set, we will employ the negative binomial model with terms for treatment group, subgroup variable, and treatment by subgroup variable to test for significance of the interaction terms. Models will be adjusted for stratification variables before treatment effects in subgroups are estimated. Forest plots will be generated displaying the estimated IRRs, and 95% CIs for each subgroup will be presented. For subgroups defined using continuous variables, analyses based on the continuous form will be considered primary, but these variables can also be categorized [111]. Non-linear effects will also be explored using splines [112]. The following subgroups determined at baseline will be examined: vascular access (ventral venous catheter and arteriovenous access); age (≥ 65 and <65 years); race categories (White, Black, and Asian; Hispanic ethnicity); diabetes mellitus (presence or absence); and anthropometric volume (Watson [113] volume < 35 L vs. ≥ 35 L). Likelihood ratio tests will be used to determine whether treatment effects vary across subgroups, followed by post hoc HTE analysis when interaction effects are significant. We will follow Wang et al.’s recommendation and calculate and report the overall probability of type I error given the number of subgroups tested [114].

Further details regarding other statistical analyses will be provided in the statistical analysis plan prior to commencing analysis.

### Methods in analysis to handle protocol non-adherence and any statistical methods to handle missing data {20c}

To minimize missingness, we will follow standard methods for increasing response (e.g., good communication, reminders, and patient incentives for timed urine collection). Data monitoring will decrease the frequency of missing data and identify data management problems. Programmers and software developers at the DCC will develop a flexible and reliable data entry system. This system will provide multiple checks for data plausibility, including variable outliers and data completeness checks. Validation checks will be implemented to coincide with skip logic. Data entry personnel will receive ongoing feedback to facilitate complete data collection and reduce errors. Real-time reports will be incorporated into the web-based data management system to allow multiple quality control and performance monitoring reports. These include reports of missing data by Clinical Center and data entry personnel. Reasons for missing data will be collected and described. All patients will be accounted for in all analyses and presentations. To account for missingness, those discontinuing the study prematurely will be censored at the time of dropout. Sensitivity analyses will be undertaken [115], including inverse probability weighting (IPW) [116, 117] under the missing at random assumption (MAR) [118, 119] and pattern-mixture approach to explore the possible effect of deviations from MAR [120, 121].

### Plans to give access to the full protocol, participant-level data, and statistical code {31c}

Data and the protocol will be available upon request and permission of relevant authorities (e.g., Principal Investigators, dialysis organization leadership, and funding agency) after the trial.

## Oversight and monitoring

### Composition of the coordinating center and trial steering committee {5d}

The study will be led by two main Principal Investigators (PIs). Wake Forest University School of Medicine (WFUSM) will be the prime institution of the TwoPlus Research Consortium with the role of CCC and will also include the Implementation Science Team (IST). The DCC will be located at New York University (NYU) Langone. Enrollment will take place at Clinical Centers of national healthcare systems and their affiliated dialysis organizations, under the leadership of Site PIs. An External Expert Advisor will provide guidance regarding the monitoring of residual kidney function and solute clearance. The TwoPlus Research Consortium has partnered two largest patient advocacy organizations in the USA to make certain the study is continuously informed by patient insights: the American Association of Kidney Patients and Home Dialyzors United. The TwoPlus Research Consortium Team has also partnered with stakeholders involved in providing hemodialysis care, i.e., clinicians, nurses, dietitians, and social workers. These Stakeholder Partners will represent the Stakeholder Advisory Panels across Clinical Centers (Additional file 1, Figure S1).

### Steering committee

The Steering Committee will be the main governing body of the study. This committee will be comprised of the main PIs, Lead Biostatistician, Site PIs, Stakeholder Partners, External Expert Advisor, and Dissemination Committee Chair. The members of the Steering Committee will (i) be responsible for the overall study governance; (ii) hold regular meetings with Stakeholder Advisory Panels; (iii) review Safety Reports and manage communications with central IRB and PCORI Project Officials; (iv) report study results; and (v) oversee return of aggregated study results to the community, decision-makers, and policy-makers. The Steering Committee will receive input from the External Expert Advisor on matters associated with scientific developments during the study. The Steering Committee will convene on a regular basis through virtual meetings and correspond often via emails.

### Dissemination committee

This committee will be formed by professional staff and organizational leaders of the American Association of Kidney Patients (AAKP). Together with the main PI, Site PIs, IST, this committee is tasked with the dissemination of study implementation, study progress, and study results. The main PIs and the Chair of the Dissemination Committee will meet on a regular basis with payor representatives of the Centers of the Medicare and Medicaid Services (CMS), primary payor for dialysis service in the USA.

### Other committees

The *Project Managers Committee* is comprised of the project managers of the CCC, DCC, and IST and is tasked with overseeing study implementation at all Clinical Centers, monitoring of recruitment, study-specific activities, and periodic retraining at each Clinical Center; and reporting of protocol deviations and serious adverse events (SAEs) to regulatory bodies. The *Publications and Presentations Committee* and the *Ancillary Studies Committee* will develop ideas for future studies and manuscripts, and they will work with the Steering Committee to identify the investigators within the consortium that can lead these efforts.

### Composition of the data monitoring committee, its role and reporting structure {21a}

An independent Data and Safety Monitoring Board (DSMB) will have the primary responsibility for monitoring the accumulating study data for signs of adverse trends in morbidity/mortality and reportable SAEs. This committee will be composed of six members, inclusive of the DSMB Chair. The DSMB includes one biostatistician, three nephrologists, one patient representative, and one caregiver representative. Meetings of the DSMB will be held periodically. Material for these meetings will be prepared by the DCC and distributed 2 weeks before the meetings via communication with the PCORI Team. The DSMB will have unlimited access to data upon request.

### Adverse event reporting and harms {22}

The TwoPlus trial poses a level 3, moderate risk for patient participants, and a level 1, no more risk than expected in daily life for caregiver and the members of the advisory panels as defined in federal regulations at 45 CFR 46.102(i) and 21 CFR 50.3(k). Expected risks include electrolyte and acid-base imbalances and volume overload, each with attendant consequences (Additional file 1, Table S4).

#### Adverse events

An adverse event (AE) is any untoward medical occurrence in a participant, which does not necessarily have a causal relationship with the study intervention. In the context of the TwoPlus trial, an AE will be considered to be:

- Any unintentional, unfavorable clinical sign or symptom, including complications of HD
- Any new illness or disease or the deterioration of existing disease or illness
- Any clinically significant deterioration in any laboratory assessments or clinical tests

In the context of the TwoPlus trial, the circumstances listed below will NOT be considered to be AEs:

- A pre-existing condition (unless it worsened significantly during HD)
- Routine diagnostic and therapeutic procedures for an incident or chronic conditions

AEs will be assessed for severity and relationship to the study.

#### Classification of an AE

All AEs will be classified using the current version of the Common Terminology Criteria for Adverse Events (CTCAE) developed and maintained by CTEP at National Cancer Institute, as follows:

- *Mild—*awareness of sign, symptom or event, but easily tolerated; requires no special treatment and does not interfere with the participant’s daily activities.
- *Moderate—*discomfort enough to cause interference with usual activity and may warrant intervention.
- *Severe—*incapacitating with inability to do usual activities or significantly affects clinical status and warrants intervention.
- *Serious (SAEs)*

#### SAEs

These are AEs that meet any of the following criteria:

- Results in death
- Is life threatening, or places the participant at immediate risk of death from the event as it occurred
- Requires or prolongs hospitalization
- Causes persistent or significant disability or incapacity
- Results in congenital anomalies or birth defects
- Is another condition which investigators judge to represent significant hazards

#### Relationship to study intervention

The Site PIs will grade the degree of certainty about causality by using the categories below.

◦ *Definitely Related to the study intervention—*There is clear evidence to suggest a causal relationship with the study intervention, i.e., randomization and assigned HD treatment group, and other possible contributing factors can be ruled out. The clinical event, including an abnormal laboratory test result, occurs in a clear, direct time relationship to study intervention administration and cannot be explained by concurrent disease or other drugs or chemicals.
◦ *Probably related to the study intervention*—An event that follows a reasonable temporal association with the study intervention, i.e., randomization and ascribed HD treatment group, that is not easily explained by another cause such as known characteristics of the participant’s clinical state or other treatment.
◦ *Probably not related to the study intervention*—An event that does not follow a reasonable temporal association with the study intervention, i.e., randomization and ascribed HD treatment group, that can be explained by another cause such as known characteristics of the participant’s clinical state or other treatment.
◦ *Definitely not related to the study intervention*—There is clear evidence that there is no causal relationship with the study intervention, i.e., randomization and assigned HD treatment group, and other possible contributing factors have been ruled in. The clinical event, including an abnormal laboratory test result, occurs in a clear, direct time relationship with other etiology and/or can be explained by concurrent disease or other drugs or chemicals.
◦ *Causal relationship/relatedness to the study intervention is not assessable*—There is insufficient or incomplete evidence to make a clinical judgement of the causal relationship.

#### Protection against risks

##### Residual kidney function

The TwoPlus EDC platform will generate alerts when (a) data needed to monitor residual kidney function are missing and (b) residual kidney function metrics indicate a patient participant can transition from twice-weekly to thrice-weekly HD.

##### Summary data

These data will comprise participants’ biochemical laboratory values and volume status management. Summary data, for each Clinical Center, will be generated monthly by the DCC and will be reviewed by the Site Investigators who, in turn, will take appropriate measures and contact the treating providers.

##### Patient participant report

Patient participants will be provided with a report on a quarterly basis or more often, when timed urine collection and residual kidney function is assessed. These reports will include a summary of the last 2 measurements of residual kidney function, pre-HD serum potassium, pre-HD serum bicarbonate, and average interdialytic weight gain over the last 2 weeks. These reports will increase the interactions between study personnel and participants, giving an opportunity to the patients to ask questions pertaining to their study participation, and questions that can be relayed to the treating team and/or study team.

### Frequency and plans for auditing trial conduct {23}

The CCC and DCC will perform audits periodically during the entire duration of the trial. At each audit, congruousness with respect to trial procedures and data collection will be evaluated. Any critical issues will be discussed by the steering committee and communicated to clinical center PIs and local study coordinators. Additionally, auditing may be performed, without anticipated communication, by the local regulatory bodies.

### Plans for communicating important protocol amendments to relevant parties (e.g., trial participants, ethical committees) {25}

All modifications about protocol and procedures must first be approved, by amendment, by the funder, the DSMB and the Steering Committee, and then by the central IRB of the WFUSM. If approved, the changes will be communicated and reported in the national web-based platform of inter-institutional reliance exchange program.

### Dissemination plans {31a}

The research team has partnered with the AAKP, the oldest and largest independent kidney patient organization in the USA. AAKP will participate in the dissemination of study results, carefully tailoring strategies to match program, research, and educational objectives and key milestones. Through its Centers for Patient Research & Education and Center Patient Engagement & Advocacy, AAKP is uniquely positioned to engage with a variety of key stakeholders, including patients, researchers, state elected, and appointed leaders as well the U. S. Congress and appointed federal officials at the White House and within federal health agencies. AAKP will use its comprehensive communications resources to achieve the highest levels of dissemination and impact possible.

The study’s findings will be distributed by its Site Investigators and Stakeholder Partners. These results are intended to be shared with the wider scientific community through publications in scholarly peer-reviewed journals and showcased at conferences via presentations and poster sessions. Following their official publication, a summary of the key findings may also be disseminated through targeted websites, forums, and various social media platforms. Neither the funder not any pharmaceutical company plays no part in the conduct, analysis, interpretation of the data, or in the sharing of the study’s outcomes.

## Discussion

### Public health impact

KDRD is a burdensome condition at individual and societal levels, both in terms of the direct costs of treatment and the associated costs of disability, dependency, and unemployment. Every day, 350 people in the USA are diagnosed with KDRD [122]. The prevalence of this condition is expected to double by 2050, driven by an annual increase in incidence rates of 8%, heralding unmanageable costs against the backdrop of strained healthcare economies and dripping resources [123]. Over 90% of people diagnosed with KDRD are treated with the same regimen of thrice-weekly, CHD [124], regardless of the seriousness of their KDRD [33, 34]. The 2019 Executive Order on Advancing American Kidney Health Initiative urged innovative interventions to transform kidney disease care and reduce costs [125, 126]. Consequently, there is a compelling demand for HD treatment modalities that offer personalized care, alleviate the burden of treatment, and reduce healthcare resource utilization, all while maintaining the quality of health-related outcomes [45]. In pursuit of this goal, it is critical to conduct research that rigorously evaluates the effectiveness of CMIHD in comparison to CHD among appropriate patient populations. The findings from such research will be pivotal in endorsing the widespread adoption of CMIHD within common clinical settings and will also provide patients and advocacy organizations additional data on a treatment option that expands their patient care choice.

### The overarching goal of the TwoPlus trial

CMIHD is a form of personalized, kidney function-coupled HD does not assume that all patients need the same minimum HD intensity conventionally delivered thrice-weekly at a minimum dialysis intensity with each treatment [5, 6]. It further recognizes that the minimum CHD intensity is also a prescription of maximum effectiveness beyond which clinical trials have shown that a higher HD dose would add little if any clinical benefit [127, 128]. Several observational studies and two pilot clinical trials indicated that CMIHD prescribed to patients with apposite levels of residual kidney function is similarly effective to CHD [22]. The absence of multicenter prospective and randomized studies to objectively assess the clinical effectiveness and practical implementation of CMIHD is contributing to the lack of systematic adoption of this treatment modality in clinical practice. Drawing from practical clinical insights [46, 64, 129, 130] and pilot clinical trial findings [131, 83, 132], the TwoPlus trial is designed to conduct a fair and thorough evaluation of patient outcomes across diverse healthcare systems and dialysis organizations, based on the initial HD schedule. Should the trial demonstrate the effectiveness of CMIHD, we anticipate that its findings will catalyze advancements, paving the way for incremental-start HD to become a universally accessible option for all patients, in all countries.

### Trial status

Protocol version 0.5, 31-01-2024

The recruitment for this study is anticipated to begin on 01 March 2024. Recruitment and data collection is anticipated to end on 01 March 2028.

### Supplementary Information

Supplementary Material 1.

Supplementary Material 2. 

Supplementary Material 3. 

Supplementary Material 4. 

## Data Availability

The DCC tracks the life cycle for all data sets to be collected, processed, and transferred in the context of the TwoPlus Trial. Substantive metadata and source code will be generated and stored with the data during this process. For ease of access, codebooks for harmonized datasets will be generated and stored alongside data files. They will be shared with other funded centers through a shared file directory accessible through the study website. Study protocol, manual of operations, training manuals, and related documents are available behind the login wall on the study website. All completed analyses, derived datasets, and metadata will be digitally archived. For data sharing, the DCC will construct a public-use version of the final research, with the contents and corresponding metadata to be determined jointly by the PI and collaborating centers.

## Abbreviations

AAKP: American Association of Kidney Patients
AE: Adverse event
ANCOVA: Analysis of covariance
CCC: Clinical Coordinator Center
CFR: Code of Federal Regulations
CHD: Conventional hemodialysis
CMIHD: Clinically Matched Incremental HD
CMS: Centers of the Medicare and Medicaid Services
CTCAE: Common Terminology Criteria for Adverse Events
DCC: Data Coordinator Center
DSMB: Data and Safety Monitoring Board
ED: Emergency department
EDC: Electronic data capture
EPOCH-RRT: Empowering Patients on Choices for Renal Replacement Therapy
ESKD: End-stage kidney disease
HD: Hemodialysis
HTE: Heterogeneity of treatment effects
IRB: Institutional Review Boards
IST: Implementation Science Team
ITT: Intention to treat
KDRD: Kidney Dysfunction Requiring Dialysis
NYU: New York University
PCORI: Patient-Centered Outcomes Research Institute
PI: Principal Investigator
RCT: Randomized controlled trial
RMANCOVA: Repeated-measures analysis of covariance
SAE: Serious adverse event
SONG-HD: Standardized Outcomes in Nephrology–Hemodialysis
WFUSM: Wake Forest University School of Medicine
