# Supplementary material for: Comparative effectiveness of an individualized model of hemodialysis vs conventional hemodialysis: a study protocol for a multicenter randomized controlled trial (the TwoPlus trial)
Source: Trials. 2024 Jun 28;25:424. doi: 10.1186/s13063-024-08281-9 (PMC11212207; doi:10.1186/s13063-024-08281-9)
Supplement: Supplementary file 1 — Supplementary Material 1. [file 13063_2024_8281_MOESM1_ESM.zip › Figure S1_Consortium Organizational StructureR1.pptx]

## Slide 1
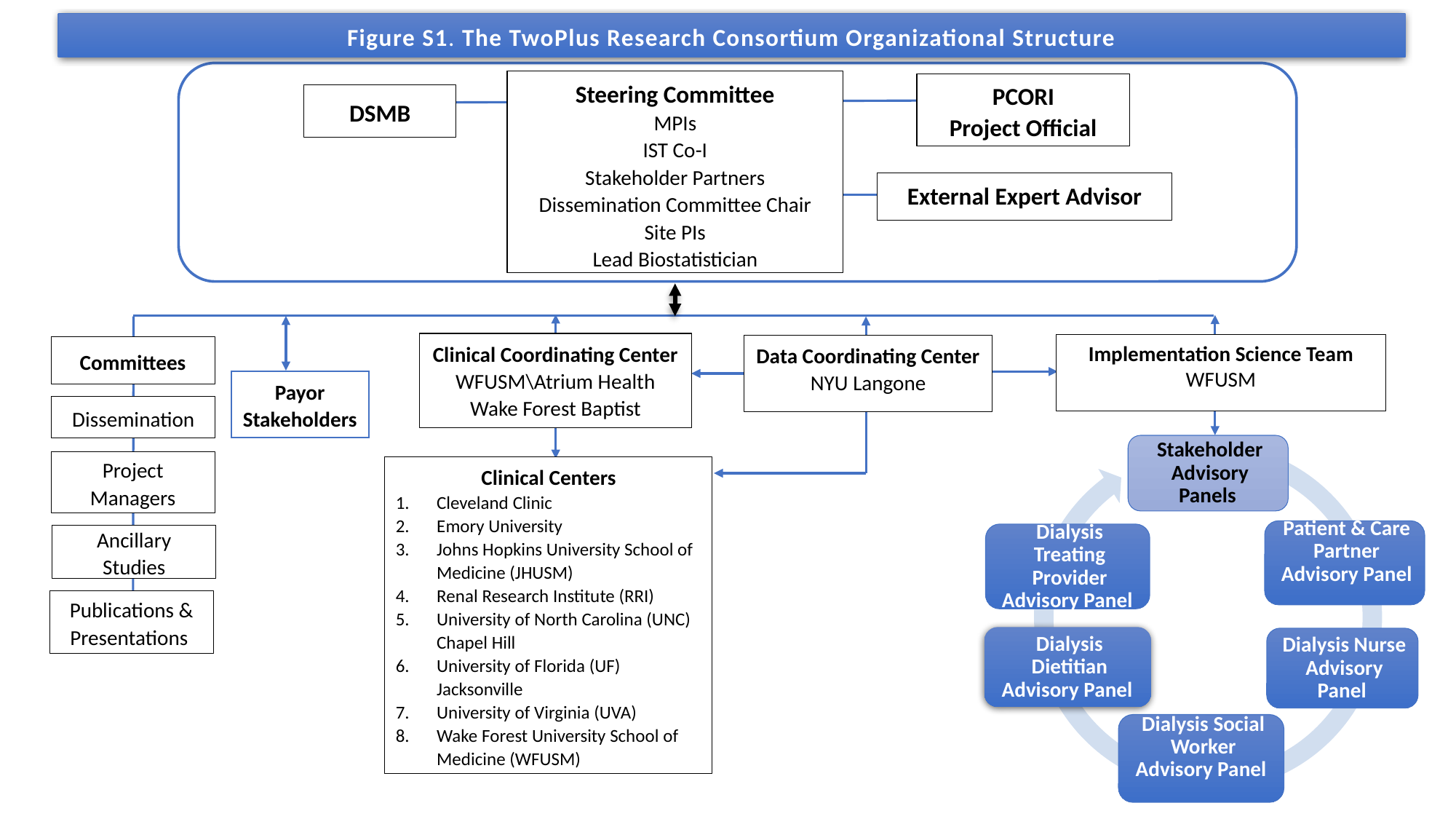

Figure S1. The TwoPlus Research Consortium Organizational Structure
Steering Committee
MPIs
IST Co-I
Stakeholder Partners
Dissemination Committee Chair
Site PIs
Lead Biostatistician
PCORI
Project Official
DSMB
External Expert Advisor
Committees
Project Managers
Ancillary Studies
Publications & Presentations
Clinical Coordinating Center WFUSM\Atrium Health Wake Forest Baptist
Implementation Science Team WFUSM
Data Coordinating Center
NYU Langone
Payor Stakeholders
Dissemination
Clinical Centers
Cleveland Clinic
Emory University
Johns Hopkins University School of Medicine (JHUSM)
Renal Research Institute (RRI)
University of North Carolina (UNC) Chapel Hill
University of Florida (UF) Jacksonville
University of Virginia (UVA)
Wake Forest University School of Medicine (WFUSM)
