# Supplementary material for: Comparative effectiveness of an individualized model of hemodialysis vs conventional hemodialysis: a study protocol for a multicenter randomized controlled trial (the TwoPlus trial)
Source: Trials. 2024 Jun 28;25:424. doi: 10.1186/s13063-024-08281-9 (PMC11212207; doi:10.1186/s13063-024-08281-9)
Supplement: Supplementary file 1 — Supplementary Material 1. [file 13063_2024_8281_MOESM1_ESM.zip › Figure S2_Recruitment and RetentionR1.pptx]

## Slide 1
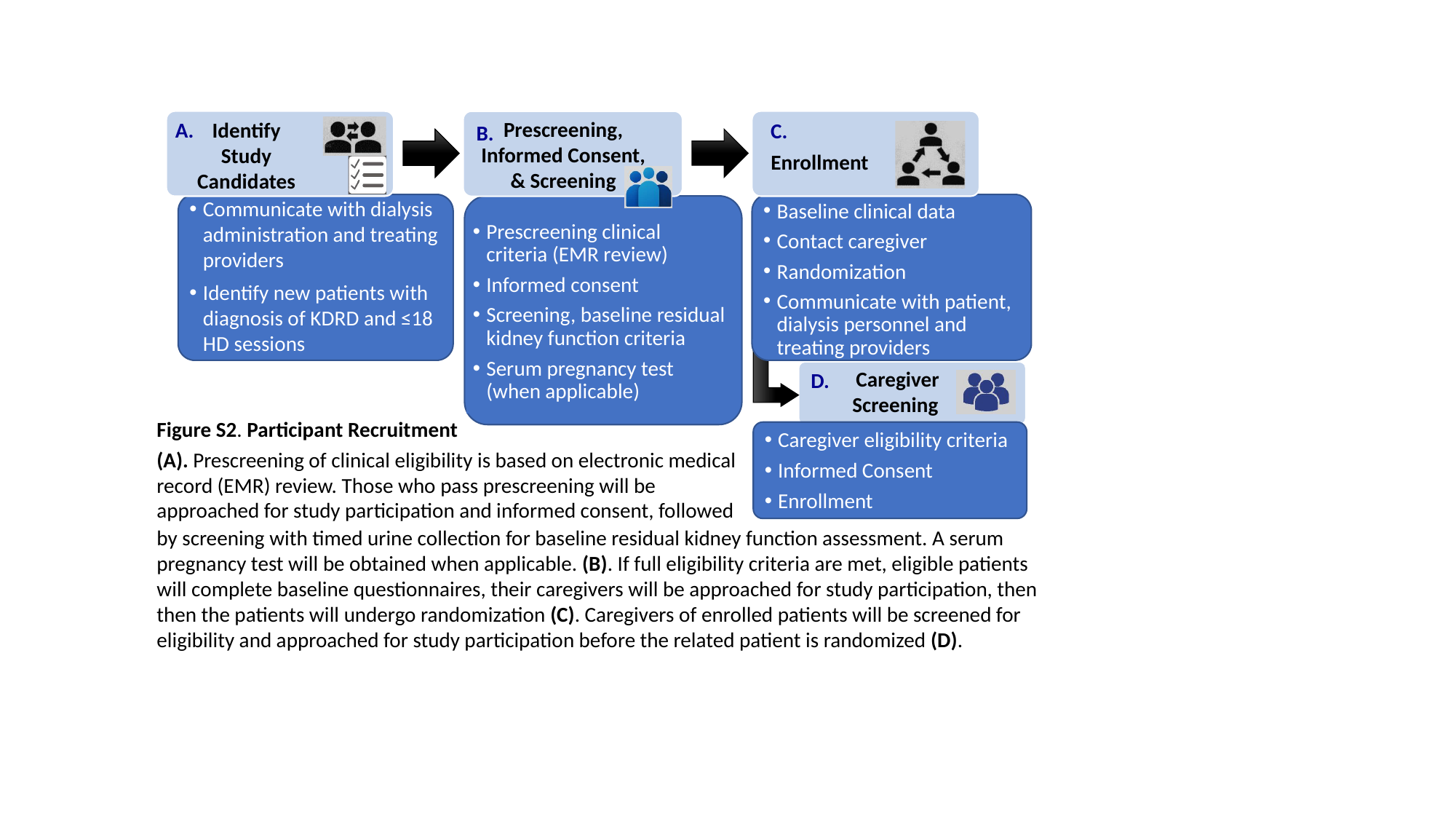

Prescreening, Informed Consent, & Screening
Identify Study Candidates
A.
C.
B.
Enrollment
Communicate with dialysis administration and treating providers
Identify new patients with diagnosis of KDRD and ≤18 HD sessions
Baseline clinical data
Contact caregiver
Randomization
Communicate with patient, dialysis personnel and treating providers
Prescreening clinical criteria (EMR review)
Informed consent
Screening, baseline residual kidney function criteria
Serum pregnancy test (when applicable)
Caregiver Screening
D.
Figure S2. Participant Recruitment
Caregiver eligibility criteria
Informed Consent
Enrollment
(A). Prescreening of clinical eligibility is based on electronic medical record (EMR) review. Those who pass prescreening will be approached for study participation and informed consent, followed
by screening with timed urine collection for baseline residual kidney function assessment. A serum pregnancy test will be obtained when applicable. (B). If full eligibility criteria are met, eligible patients will complete baseline questionnaires, their caregivers will be approached for study participation, then then the patients will undergo randomization (C). Caregivers of enrolled patients will be screened for eligibility and approached for study participation before the related patient is randomized (D).
