# Supplementary material for: Comparative effectiveness of an individualized model of hemodialysis vs conventional hemodialysis: a study protocol for a multicenter randomized controlled trial (the TwoPlus trial)
Source: Trials. 2024 Jun 28;25:424. doi: 10.1186/s13063-024-08281-9 (PMC11212207; doi:10.1186/s13063-024-08281-9)
Supplement: Supplementary file 1 — Supplementary Material 1. [file 13063_2024_8281_MOESM1_ESM.zip › Tables S1 to S4R1.docx]

| **Table S1**. **Lab Tests by Randomized Treatment Group** | | |
| --- | --- | --- |
| **Variable** | **CMIHD (intervention group)** | **CHD (control group)** |
| Chemistry, serum:  Na, K, TCO2 | At least once a month. Per usual care, these are obtained at least once a month, sometimes more often. Frequency of lab checks may increase at the decision of the site investigators and\or treating providers. | Per local, usual care |
| B2MG, serum | When timed urine collection is submitted. | When timed urine collection is submitted. |
| Timed urine collection: urine volume, urine urea nitrogen, urine creatinine | Every 3 months from the month of baseline (screening) residual kidney function.And when deemed medically necessary. | Every 3 months from the month of baseline (screening) residual kidney function.  And when deemed medically necessary. |
| *Other blood tests, obtained as part of usual care at outpatient HD units, will be collected in both treatment group at a frequency according to local care. These include: BUN; CBC; dialysis urea clearance (spKt/V and URR); serum creatinine; serum phosphorus; serum calcium; serum intact parathyroid hormone (iPTH); serum ferritin; serum transferring saturation. BUN, blood urea nitrogen; B2MG, beta 2 microglobulin; CBC, complete blood cell count; K, potassium; Na, sodium; spKt/V, single pool Kt/V urea; TCO2, bicarbonate; URR, urea reduction ratio.* | | |

| **Table S2**. **Sample Size (N) Required for Proving Non-Inferiority in Primary Composite Outcome** | | | | | | | | | |
| --- | --- | --- | --- | --- | --- | --- | --- | --- | --- |
| Detectable Non-Inferiority Margin | Estimated Incidence Rate Ratio (IRR)^#^ | | | | | | | | |
|  | 0.95 | | | 0.90 | | | 0.85 | | |
|  | 80% Power | 85% Power | 90% Power | 80% Power | 85% Power | 90% Power | 80% Power | 85% Power | 90% Power |
| 1.20 | 456 | 520 | 608 | 308 | 350 | 410 | 218 | 250 | 294 |
| 1.25 | 330 | 378 | 440 | 236 | 270 | 316 | 176 | 200 | 236 |
| 1.30 | 254 | 290 | 338 | 188 | 216 | 254 | 146 | 166 | 194 |
| *N is total sample size. Sizes are assumed to be equal (N/2) between the two treatment groups. ^#^Estimates for IRRs for primary composite outcome, between CMIHD and CHD treatment groups, are based on the US pilot trial (Murea, AJKD), the UK pilot trial (Vialr, Kidney Int), and national registry data. | | | | | | | | | |

| **Table S3**. **Estimated Effect Size That Can Be Detected for Secondary Outcomes** | | | |
| --- | --- | --- | --- |
| Secondary Outcome (baseline-adjusted) | SD at baseline | Average difference detected between groups | |
|  | Baseline | 80% power | 90% power |
| Illness Intrusiveness Rating Scale score | 16.2 | 3.26 | 3.78 |
| EuroQOL-5 Dimensions-5 Level score | 0.245 | 0.05 | 0.06 |
| Change in urine volume (mL/24 h) | 955 | 192 | 222.5 |
| Change in kidney urea clearance (mL/min/1.73 m^2^) | 2.4 | 0.48 | 0.56 |
| Change in kidney creatinine clearance (mL/min/1.73 m^2^) | 6.3 | 1.27 | 1.47 |
| Zarit Caregiver Burden Scale | 9.38 | 1.89 | 2.19 |
| Note: Power estimated at an average follow-up of 2 years per study participant. | | | |

| **Table S4**. **Potential Safety Risks and Procedures to Monitor Safety** | | | | |
| --- | --- | --- | --- | --- |
| **Risk** | **Consequence** | **Assessment** | **Assessment Frequency in Usual Practice** | **Summary Data Reviewer** |
| Occult loss of residual kidney function | Suboptimal solute clearance; Symptoms of uremia (e.g., nausea, vomiting, poor appetite, altered mental status);  Hyperkalemia;  Metabolic acidosis;  Arrhythmia | Clinical exam | At least once a month by the provider physician. And, at least once a month by other treating providers (e.g., APP). And at every HD treatment by dialysis nurse personnel. | Treating providers & dialysis personnel |
|  |  | Timed urine collection | Minimum every 3 months (from the month of baseline residual kidney function) when clinically stable. Within 2-4 weeks after an acute illness that could have affected residual kidney function. More often when deemed clinically necessary. | Study team & Treating providers |
|  |  | Basic metabolic panel | Every 4 weeks (or more often, per Site Investigators or treating providers). | Study team & Treating providers |
|  |  | stdKt/V urea, kidney and dialysis | Monthly or more often as medically necessary. | Study team & Treating providers |
| Extracellular volume overload | Uncontrolled HTN; Chest pain; Shortness of breath;  Hospitalization related to volume overload or cardiovascular events* | Clinical exam | At least once a month by the provider physician. And, at least once a month by other treating providers (e.g., APP). And at every HD treatment by dialysis nurse personnel. | Treating providers and dialysis personnel |
|  |  | Metrics of volume management | Monthly | Study team & Treating providers |
|  |  | DSMB meeting | Semi-annual | DSMB |
| Protocol non-adherence | Suboptimal solute clearance | Clinical exam | At least once a month by the provider physician. And, at least once a month by other treating providers (e.g., APP). And at every HD treatment by dialysis nurse personnel. | Study team & Treating providers & dialysis personnel |
|  |  | HD frequency | According to usual care, four times per month at the outpatient dialysis center. | Study team & Treating providers |
| *As deemed by the Site Investigators and/or treating providers. APP, advanced practice provider; DSMB, Data and Safety Monitoring Board; HTN, hypertension; stdKt/V, standard Kt/V urea. | | | | |
