# Supplementary material for: Comparative effectiveness of an individualized model of hemodialysis vs conventional hemodialysis: a study protocol for a multicenter randomized controlled trial (the TwoPlus trial)
Source: Trials. 2024 Jun 28;25:424. doi: 10.1186/s13063-024-08281-9 (PMC11212207; doi:10.1186/s13063-024-08281-9)
Supplement: Supplementary file 2 — Supplementary Material 2. [file 13063_2024_8281_MOESM2_ESM.docx]

# Patients: Notes Regarding Eligibility Criteria

*The decision for study eligibility (i.e., eligibility for twice-weekly HD) should be made by the treating nephrologist in conjunction with the Site Investigators.

¥ The time when the patient is approached for potential study participation refers to the date when the patient is approached for getting an informed consent for study participation (i.e., the date when a member of the study team discusses with the patient about the study and offers study participation). All efforts must be made to assess eligibility and recruit as early as possible after HD initiation, ideally within 1-2 weeks of HD commencement. For patients who are anticipated to be started on maintenance, in-center HD, decision of HD initiation is made by the treating providers.

¶ Both parameters of residual kidney function (kidney urea clearance and urine volume) must be met to be eligible for study participation.

§ Examples of high-volume ultrafiltration requirement: interdialytic weight gain ≥ 4% of target weight; post-HD residual weight ≥5% of target weight.

**If abnormal values, reassess at a later date (e.g., after 12 HD sessions, sooner, or later) provided patient would not have been on HD for >6 weeks (i.e., first date of HD was not >42 days prior) at the time patient will be approached for potential study participation; and target weight was dynamically reassessed and gauged by the Site Investigators and treating providers.

€ Examples given: urinary incontinence; severe dementia; history of medical non-adherence that, in the opinion of the Site Investigators and/or treating providers, precludes safe study participation; a medical condition that, in the opinion of the Site Investigators and/or treating providers, would jeopardize the safety of the participant; or unable or unwilling to give informed consent for study participation.

# If results of baseline residual kidney function levels (calculated on baseline timed urine collection) are available through chart review, they are taken into consideration for eligibility criteria if the urine collection occurred within 2 weeks prior to the prescreening date. If more than 2 weeks elapsed, then recruitment should follow scenario a), i.e., a new baseline timed urine collection will be obtained at the time of screening, after informed consent for study participation is obtained.

‡ We define women of childbearing capacity as women age ≤55 years old who do not have a history of hysterectomy (see below for more details).

Kidney dysfunction requiring dialysis (KDRD) connotes the same concept as end-stage kidney disease (ESKD) or end-stage renal disease (ESRD) or chronic kidney disease stage 5 on dialysis (CKD5D).

The decision to initiate chronic, in-center HD is made by the treating providers, independent of this study.

Target weight connotes the same concept as Dry Weight; the terminology is used interchangeably.

Calculation of Inter-dialytic Weight Gain (IDWG) as % of Target Weight:

IDWG = [(pre-HD weight – target weight)/target weight] x 100

Calculation of Residual Weight as % of Target Weight:

Residual weight = [(post-HD weight – target weight)/target weight] x 100

# Pregnancy-related eligibility assessment ‡: Patients

## Definition: woman of childbearing capacity

A woman of childbearing capacity is a person of female sex, age ≤55 years, and with presence of an anatomical uterus.

For women aged 55 or under, the sole circumstance in which they're considered to lack childbearing capacity is if the Electronic Medical Records (EMR) within the healthcare system document a history of undergoing a total hysterectomy.

*Note*: A history of total hysterectomy must be evidenced in healthcare system EMR. Scenario when history of hysterectomy is reported by the patient but not identified in the healthcare system EMR will be considered childbearing capacity.

*Note*: women age ≤55 years with either i) reported amenorrhea, or ii) tubal ligation, or iii) intrauterine device, or iv) on contraceptive methods are considered of childbearing capacity.

## Approach to pregnancy-related eligibility assessment

Evaluation for study eligibility, from the viewpoint of pregnancy, will take place at 3 levels for all women ≤55 years:

1. At prescreening: through review of EMR;
2. At Informed Consent: through specific conversations between study team member and patient; and
3. Serum Pregnancy Test: blood test, either qualitative or quantitative serum pregnancy test, obtained after informed consent is signed.

# Caregiver\Care partner: Note Regarding Eligibility Criteria

*Note for caregiver eligibility #d): Examples of psychiatric and neurologic disorders include: bipolar disorder; severe depression requiring hospitalization; schizophrenia; delusional disorder; stroke with residual functional impairment such as hemiplegia, paraplegia, or hemiparesis.

# Notes for Table 3: Criteria for Progression from Twice-weekly to Thrice-weekly HD

*The decision to transition from twice- to thrice-weekly HD is made by the treating nephrologist in conjunction with the Site Investigator, not by a study coordinator. No criterion, taken in isolation, is an absolute indication for transitioning from twice- to thrice-weekly HD. Each criterion must be judged in the overall clinical context for each individual to decide on medical necessity and timing of transition from twice- to thrice-weekly HD.

^€^Either one parameter of residual kidney function (i.e., either kidney urea clearance or urine volume) or both parameters can qualify the patient to transition from twice- to thrice-weekly HD.

^#^ On one or two determinations.

^¥^Residual kidney function may be re-assessed within 2 weeks of antibiotic treatment completion, to evaluate whether conversion back to twice-weekly HD is acceptable, provided no other indication exists for thrice-weekly HD.

Inter-dialytic weight gain % calculated as [(pre-HD weight – target weight)/target weight] x 100.

Residual weight calculated as [(post-HD weight – target weight)/target weight] x 100.

# Notes for Table 6: Time Schedule of Assessment for Patients and Caregivers Enrolled in the TwoPlus Trial Baseline residual kidney function parameters: urine volume, urine urea nitrogen, and (pre-HD and post-HD) BUN.

Follow-up residual kidney function parameters obtained at least every 3 months from the month of baseline residual kidney function assessment that was obtained at the time of screening: urine volume, urine urea nitrogen, urine creatinine; and pre-HD and post-HD BUN; pre-HD serum creatinine, and pre-HD serum B2MG.

^a^ Based on timed urine collection: urine volume (mL per duration of collection), kidney urea clearance, kidney creatinine clearance.

^b^ Obtained as part of usual care at outpatient HD units.

^c^ First 2 weeks of the month, via telephone.

^#^ For those who have residual kidney function parameters available at prescreening stage, results of the residual kidney function tests will be taken into account for eligibility purposes if the tests (urine volume per day and kidney urea clearance) were done within 2 weeks prior to the date of prescreening.

† From each HD treatment administered at the outpatient HD unit and during hospitalization.

¶ Timed urine collection; for patients on HD, it is preferred that the date of urine collection completion to be on a same date of the week as when monthly blood tests done as part of usual care at outpatient HD units.

^€^ No less often than every 3 months; can be done more often depending on clinical assessment.

# Participant timeline: additional information

A series of baseline questionnaires and patient-reported data will be obtained before randomization. Month 0 is the baseline month of assessment and will correspond to the month when baseline (screening) residual kidney function was assessed. Month 0 visit period start date is the date of Informed Consent, and visit period end date is the last date of the month when screening residual kidney function was assessed. For each follow-up month (Month 1 to end of study), the visit period start date is the first date of the follow-up calendar month and visit period end date is the last date of the follow-up calendar month. During this follow up period, patients will have monthly blood tests done according to usual care at outpatient dialysis units. As part of the study protocol, patients will have monthly questionnaires, quarterly (or more frequent when medically deemed necessary) assessments of residual kidney function and pre-dialysis serum beta 2 microglobulin, and semiannual assessments of their cognitive function.

# Recruitment timeline: additional information

The period from the initial prescreening to obtaining informed consent is set at a maximum of 6 weeks or 18 hemodialysis (HD) treatments following the patient's initiation of their first HD session. Within the timeframe extending from the acquisition of informed consent to the date of randomization, we will measure the baseline residual kidney function and perform a serum pregnancy test, when relevant. The study teams will aim for efficiency in patient eligibility evaluation during the interval between informed consent and randomization, targeting a period of less than 6 weeks in the following manner: completion of the screening timed urine collection within 1-2 weeks post-informed consent; completion of caregiver enrollment (when applicable) within 1 week after the patient meets screening eligibility requirements; and subsequent patient randomization ideally falling within 2 weeks of baseline timed urine collection.
