# Supplementary material for: Comparative effectiveness of an individualized model of hemodialysis vs conventional hemodialysis: a study protocol for a multicenter randomized controlled trial (the TwoPlus trial)
Source: Trials. 2024 Jun 28;25:424. doi: 10.1186/s13063-024-08281-9 (PMC11212207; doi:10.1186/s13063-024-08281-9)
Supplement: Supplementary file 3 — Supplementary Material 3. [file 13063_2024_8281_MOESM3_ESM.docx]

# Patient Informed Consent for Study Participation

This will be obtained in writing and in-person by a member of the study team; and encompasses options for the following three levels of participation: ***i)*** to enroll and undergo randomization along with attendant follow-up assessments should eligibility be confirmed based on residual kidney function obtained at baseline; ***ii)*** to participate in semi-structured interviews; and ***iii)*** to allow the study team contact their care partner, should the patient identify an individual as being their care partner.

# Patient Participant Informed Consent for Limited Data Collection

In the following scenarios, the study team will seek a new signed, in-person patient informed consent to continue collection of limited data:

1. Patient participant expressed desire to withdraw consent from participating in the study in the form of providing serial timed urine collection, and dialysis modality continues to be in-center hemodialysis
2. Patient participant has dialysis modality converted to peritoneal dialysis
3. Patient participant has dialysis modality converted to home hemodialysis

Limited Data Collection will consist of elements present in the dialysis data download which will be obtained in all participants on a regular basis.

Limited Data Collection will include data that is present in dialysis EMR and healthcare system EMR, including ED visits, Hospitalizations and Death. If the patient participant will refuse to grant continued collection of limited data, then the patient will be removed from the study and data collection will be censored.

If the patient participant will grant continued collection of limited data, only dialysis data download collection will be continued until an end-of-study event.

# Caregiver Informed Consent.

This will be obtained via telephone by a member of the study team from one caregiver of the patient participant, and will have two options of participation: ***i)*** to enroll in the study and undergo questionnaire administration semi-annually; and ***ii)*** to participate in semi-structured interviews.

There will be only one caregiver per patient participant enrolled in the study, at any single time point. In the event of a drop out event for a caregiver participant occurs, this will be recorded accordingly in the EDC system and the study team will not seek enrollment of a different caregiver for the respective patient participant, even if the patient has had more than one caregiver.

# Stakeholder Advisory Panel Member Informed Consent.

Depending on the type of study activity, there will be two types of consent that will be sought from stakeholders:

1. Electronical consent, at the time of survey distribution via REDCap-linked encrypted emails;
2. Telephone consent, obtained by a member of the study team, with each semi-structured interview.

From one study activity to another, the members of the stakeholder advisory panels may change. For example, the stakeholders who participated in survey, may or may not choose to participate in semi-structured interviews. Similarly, if a stakeholder participated in a semi-structured interview, they may or may not choose to participate in another semi-structured interview at a later date. Telephone consent will be obtained, by a member of the study team and from the stakeholder, for each semi-structured interview, regardless of whether or not the stakeholder respondent participated in a prior interview.

Waiver of in-person, signed consent for caregiver and provider participation

Waiver of in-person, signed consent for caregiver and provider participation is based on the fact that the research presents no more than minimal risk of harm to caregivers and providers; their participation does not involve any interventions or procedures performed on themselves; their participation involves answering questionnaires to which they may opt to answer fully or partly as they choose; there is an appropriate alternative mechanism for documenting that informed consent was obtained; and the research meets the regulatory criteria of 45 CFR 46.117(c) and 21 CFR 50.109(c). Additionally, attempt at obtaining in-person, signed informed consent from caregivers and providers would cause their participation to be unachievable given study team’s available resources would not be able to cover the wide geographical area of study implementation with travelling to all dialysis facilities and multiple time points to obtain signed consent from multiple and varied stakeholders.
