# Supplementary material for: Comparative effectiveness of an individualized model of hemodialysis vs conventional hemodialysis: a study protocol for a multicenter randomized controlled trial (the TwoPlus trial)
Source: Trials. 2024 Jun 28;25:424. doi: 10.1186/s13063-024-08281-9 (PMC11212207; doi:10.1186/s13063-024-08281-9)
Supplement: Supplementary file 4 — Supplementary Material 4. [file 13063_2024_8281_MOESM4_ESM.docx]

**CALCULATE KIDNEY UREA CLEARANCE: PATIENT DID NOT START DIALYSIS**

Urine Urea Nitrogen x Volume Urine

Kidney Urea Clearance (KUC) (mL/min) =

1.075 x BUN x Urine Duration

*Inputs:*

- Urine Urea Nitrogen in mg/dL (reported by the lab)
- Volume Urine in mL
- Urine Duration, in minutes, based on Start Date/Time and End Date/Time of urine collection

**CALCULATE KIDNEY UREA CLEARANCE: PATIENT STARTED DIALYSIS**

Urine Urea Nitrogen x Volume Urine

Kidney Urea Clearance (KUC) (mL/min) =

R x Pre-HD BUN x Urine Duration

*Inputs:*

- Urine Urea Nitrogen in mg/dL (reported by the lab)
- Volume Urine in mL
- Urine duration, in minutes, calculated from Start Date/Time and End Date/Time of urine collection
- Pre-HD BUN in mg/dL
- R = 1.075 – (0.0038 x URR – 0.070) x (Duration Urine Collection)/Duration Interdialytic Interval
- URR as a unit number (ex, URR is 75%, then enter 75 in the equation to calculate R)

*Explanations:*

- Pre-HD BUN is BUN obtained pre-dialysis, on the day when urine collection is brought
- Post-HD BUN is BUN obtained post-dialysis, on the day when urine collection is brought
- URR (example: if 75%, then enter 75 in the above formula).
  - URR = [(Pre-HD BUN – Post-HD BUN) / Pre-HD BUN] x100
- Duration of interdialytic interval (IDI), min. This is calculated as days (in minutes) between HD sessions that border the day when the urine was collected, minus the duration of HD treatment (in minutes) on the day when urine collection is brought. (example, MWF schedule, collection done Sunday, HD duration 240min, duration of interdialytic days in min is [3 days = 1440 x 3], calculate IDI = 4,320 -240min = 4080 min). (example, MWF, collection done on Tue-to-Wed, HD duration 3.5hours=210 min, , duration of interdialytic days in min is [2 days = 1440 x 2 ], calculate IDI = 1440 x 2 – 210 = 2880 – 210 = 2670)

**Convert Kidney Urea Clearance into mL/min/1.73 m^2^**

Kidney Urea Clearance (mL/min/1.73 m^2^) = [Kidney Urea Clearance mL/min x 1.73] / BSA

**CALCULATE Kidney stdKt/V**

KUC x 10,080

Adjusted V x 1,000

Kidney stdKt/V =

*Input***:**

- KUC in mL/min
- Adjusted V (L)

**CALCULATE Dialysis stdKt/V**

**Calculate spKt/V**

Td

60

Pre-HD weight – Post-HD weight

Post-HD weight

Post-HD BUN

Pre-HD BUN

Post-HD BUN

Pre-HD BUN

spKt/V = -ln ( – 0.008 x ) + (4 – 3.5 x ) x

*Inputs:*

- Pre-HD BUN (mg/dL)
- Post-HD BUN (mg/dL)
- Td is dialysis duration expressed in minutes
- Pre-HD weight (in kilograms)
- Post-HD weight (in kilograms)

**Calculate eKt/V**

Td

Td + 30.7

eKt/V = spKt/V x

*Inputs:*

- spKt/V
- Td is dialysis duration expressed in minutes

**Calculate Leypoldt Dialysis stdKt/V without UF**

10,080 x (1-e^-eKt/V^)

Td

Dialysis stdKt/V =

1-e^-eKt/V^

eKt/V

10080

N* Td

- 1

+

*Inputs:*

- eKt/V
- Td is dialysis duration expressed in minutes
- N is number of HD treatments per week

**Calculate Dialysis stdKt/V taking into account UF (Daugirdas methodology)**

S

Dialysis stdKt/V =

1 - (0.74/N x [UFW/V])

**Inputs:**

- S is Dialysis stdKt/V from the above equation (Leypoldt Dialysis stdKt/V without UF)
- N is number of HD treatments per week
- UFW is weekly fluid gain between HD sessions, i.e., total fluid removed in mL over one week
- V is adjusted V, expressed in mL

**Calculate Total stdKt/V** (target a total stdKt/V of 2.3 to achieve a minimum of 2.1)

Total stdKt/V = Dialysis stdKt/V + Kidney stdKt/V

**CALCULATE KIDNEY CREATININE CLEARANCE**

**Kidney Creatinine Clearance** (result in mL/min)

Urine Creatinine x Volume Urine

Kidney Creatinine Clearance (mL/min) =

R x Pre-HD Serum Creatinine x Urine Duration

*Inputs:*

- Urine Creatinine in mg/dL
- Volume Urine in mL
- R = 1.075 – (0.0030 x 0.88 x URR – 0.020) x (Duration Urine Collection)/Duration Interdialytic Interval
- Urine duration, in minutes, calculated from Start Date/Time and End Date/Time of urine collection
- Pre-HD Serum Creatinine in mg/dL

**Normalize Kidney Creatinine Clearance in mL/min to BSA 🡪 Kidney Creatinine Clearance mL/min/1.73m^2^**

Normalized Kidney Creatinine Clearance = (Kidney Creatinine Clearance in mL/min x 1.73) / BSA

**CALCULATE Estimated RKF (eRKF) BASED ON SERUM B2MG**

**Formula 1** (Journal of Nephrology (2021) 34:473–481)

102.419

45.150

+

eRKF (mL/min/1.73m^2^) =

+ (0.623 x sex) – 8.733

Pre-HD Serum B2MG^0.5^

Pre-HD Serum Creatinine^0.5^

+ (0.037 x Pre-HD BUN) – (0.029 x age)

**Inputs:**

Men sex = 1

Female sex = 0

Age, years

Pre-HD Serum B2MG in mg/L

Pre-HD BUN in µmol/L

Pre-HD Serum Creatinine must be in µmol/L

Pre-HD Serum Creatinine must be in µmol/L in the above formula. We must convert from mg/dL to µmol/L

Serum Sreatinine µmol/L = Serum Sreatinine mg/dL x 88.42

Pre-HD BUN must be in mmol/L in the above formula. We must convert from mg/dL to mmol/L.

BUN mmol/L = BUN mg/dL x 0.3571

**Formula 2** (PLoS ONE 10(12): e0143813.)

4.2

-

1

Pre-HD Serum B2MG

160.3 x

eRKF (mL/min/1.73m^2^) =

**Inputs:** Pre-HD Serum B2MG in mg/L

**Formula 3** (Tariq Shafi formulas, KI 2016)

Clearance urea, creatinine (mL/min/1.73m2) = 2.4 x Pre-HD BUN^0.984^ x Pre-HD serum creatinine^-1.868^

Where Pre-HD BUN and Pre-HD serum creatinine are in mg/dL

**Formula 4** (Tariq Shafi formulas, KI 2016)

Clearance urea, creatinine (mL/min/1.73m2) = 2852 x Pre-HD serum B2MG^-2.417^ x 1.592 if male

Where Pre-HD BUN and Pre-HD serum creatinine are in mg/dL and pre-HD serum B2MG is in mg/L.

**OTHER FORMULAS:**

**Calculate BSA**

BSA = 0.007184 x Height^0.725^ x Weight^0.425^

*Inputs:*

- Height in cm
- Weight (dry weight in kg)

**Calculate V** (result is given in L) (Watson formulas)

Men: V = 2.447 + 0.3362 x weight + 0.1074 X height – 0.09516 x age

Women: V = **-**2.097 + 0.2466 x weight + 0.1069 x height

*Inputs:*

- Sex
- Weight (post-dialysis weight in kg)
- Height (cm)
- Age (years)

**Calculate Adjusted V** (result is given in L)

Adjusted V = V x 0.9

**Ultrafiltration (UF) Rate [mL/kg/hour]**

UF rate is calculate per each HD treatment.

UF rate = [(Pre-HD weight – Post-HD weight) x 1000] : Dry weight : HD treatment duration

*Inputs*:

Weights in Kg

(Pre-HD weight – Post-HD weight) x 1000 --> converts the first part of the equation into mL

HD treatment duration: in hours.

**INTERDIALYTIC WEIGHT GAIN (IDWG), % of TW**

IDWG is expressed in percentage (%) of the Target weight

IDWG is calculated between 2 consecutive HD treatments.

IDWG = [(pre-HD weight of current HD treatment – post-HD weight of previous HD treatment)/target weight] x 100.

*Inputs:*

Weights in kg

Pre-HD weight of current HD treatment

Post-HD weight of the HD treatment immediately preceding the current HD treatment

**RESIDUAL WEIGHT**

RW is expressed in percentage (%) of the Target weight.

RW is calculated per each HD treatment..

RW = [(post-HD weight – target weight)/target weight] x 100.

*Inputs:*

Weights in kg
